# Circulating RNA in Kidney Cancer: What We Know and What We Still Suppose

**DOI:** 10.3390/genes12060835

**Published:** 2021-05-28

**Authors:** Alessandra Cinque, Riccardo Vago, Francesco Trevisani

**Affiliations:** 1 Urological Research Institute, San Raffaele Scientific Institute, 20132 Milano, Italy; cinque.alessandra@hsr.it (A.C.); vago.riccardo@hsr.it (R.V.); 2 Department of Urology, Università Vita-Salute San Raffaele, 20132 Milano, Italy; 3 Unit of Urology, San Raffaele Scientific Institute, 20132 Milano, Italy

**Keywords:** renal cell carcinoma, biomarkers, liquid biopsy, non-coding RNA

## Abstract

Renal cancer represents the 7th most common tumor worldwide, affecting 400,000 people annually. This malignancy, which is the third most frequent cancer among urological diseases, displays a completely different prognosis if the tumor is detected in the early stages or advance phases. Unfortunately, more than 50% of renal cancers are discovered incidentally, with a consistent percentage of cases where the tumor remains clinically silent till the metastatic process is established. In day-to-day clinical practice, no available predictive biomarkers exist, and the existent imaging diagnostic techniques harbor several gaps in terms of diagnosis and prognosis. In the last decade, many efforts have been reported to detect new predictive molecular biomarkers using liquid biopsies, which are less invasive in comparison to renal biopsy. However, until now, there has been no clear evidence that a liquid biopsy biomarker could be relevant to the creation of a precise and tailored medical management in these oncological patients, even though circulating RNA biomarkers remain among the most promising. Given the idea that liquid biopsies will play a future key role in the management of these patients, in the present review, we summarize the current state of circulating RNA (miRNA, lncRNAs, and circRNAs) as possible biomarkers of renal cancer presence and aggressiveness in patients.

## 1. Introduction

Renal cell carcinoma (RCC) in adults comprises a heterogeneous group of tumors derived from renal tubular epithelial cells and accounts for 3% of all malignancy scenarios [1], with over 400,000 new cases of RCC worldwide annually [2]. RCC shows a 1.5:1 male to female ratio, with a peak incidence between 60–70 years old [2]. The most recognized risk factors are related to smoking, obesity, hypertension, diabetes, chronic kidney disease, exposure to toxic compounds, abuse of analgesics, genetic predispositions, and hereditary syndromes [2,3]. According to the World Health Organization, there are three major histological subtypes of RCC, all differentiated by histological and molecular genetic alterations: clear cell RCC (70–80% of cases), papillary type 1 and 2 (10–15%), and chromophobe (4–5%) [4]. The gold standard treatment for RCC is represented by radical and partial nephrectomy, whereas active surveillance is suggested in the case of small and localized tumors affecting patients with multiple comorbidities [5,6,7]. Systemic treatment with immunotherapy and targeted therapy has revolutionized the oncological approach in several categories of patients with advanced metastatic cancers, even though the rate of death remains high [8]. In fact, the prognosis of this urological malignancy is closely related to the aggressiveness at the time of diagnosis. If the 5-year disease-specific survival rate for a localized and low-risk RCC (N0, M0 according to TNM classification) achieves more than 90%, a metastatic high-risk (N2, M0/M1) neoplasm may be fatal rapidly, despite surgical and oncological treatments [9,10]. For these reasons, the diagnostic process remains one of the most important clinical steps in the management of RCC patients. Unfortunately, RCC is characterized by the presence of nonspecific symptoms (blood hypertension, anemia, and weight loss) during its development in the human body, from the early stages to the advanced ones [11]. For this reason, the majority of renal tumors are diagnosed incidentally by an abdominal ultrasound or computed tomography (CT) scan performed for other medical purposes. In particular, only a CT scan and MRI allow for an accurate diagnosis of RCC, although they have some limitations in terms of distinguishing between benign and malignant neoplasms [11].

Percutaneous renal tumor biopsy can be useful for detecting the presence of an RCC or a benign neoplasm; however, due to the RCC wide heterogenicity, renal biopsies display a low accuracy in terms of histological grading [12]. Moreover, renal parenchyma has a more fragile and more vascular biological structure, which can promote hemorrhages, hematoma, and infections in a nonnegligible percentage of cases during biopsies [13]. Therefore, there is still a strong need for a new molecular tool that is able to detect, in a noninvasive way, both the presence of a renal mass and its aggressiveness in day-to-day clinical practice. In the last decade, several studies have proposed different tools for revealing RCC establishment in humans; however, none of the investigated molecular targets has changed the diagnostic algorithm in renal cancer based on a CT scan or MRI imaging. One of the most intriguing protagonists in the RCC biological biomarker scenarios is represented by the complex and various universe of the “non-coding RNA molecules” [14,15]. It is well known in the literature that the majority of human RNA transcripts do not encode for proteins and that non-coding RNAs can control cell physiology and also cellular functions [16,17]. Advances in sequencing technologies have been conducted to discover a multitude of non-coding RNA (ncRNA) categories, with some highly conserved, such as microRNAs (miRNAs), transcribed ultra-conserved regions, and circular RNAs (circRNAs), and others generally lacking conservation across species, such as long ncRNAs (lncRNAs) [18]. MiRNAs are small non-coding single-stranded RNA molecules of approximately 22 nucleotides in length, which have a crucial role in the regulation of gene expression and are consequently involved in many biological processes [14]. MiRNAs bind to the 3′ untranslated region (UTR) of their target mRNA transcripts and cause their translational repression if there is an imperfect complementarity between the miRNA and the target mRNA sequence or their degradation in the case of a perfect complementarity [19]. Each miRNA can regulate up to 100 different mRNAs, and more than 10,000 mRNAs seem to be directly regulated by miRNAs [20]. There is increasing evidence that miRNAs can play a key role in tumorigenesis, tumor progression, and metastasis. MiRNAs act as oncogenes (OncomiRs) and downregulate tumor suppressor genes, and they are frequently overexpressed in cancer, while tumor suppressor miRNAs downregulate oncogenes and are often under-expressed in cancer [21,22]. These characteristics make them excellent candidates as biomarkers not only for the diagnosis, but also for the prognosis and the classification of cancer [23]. LncRNAs are a group of untranslated, regulatory RNA longer than 200 nucleotides. So far, they have been reported to function through multiple mechanisms that have prominent roles in cancer, including interactions with both genes and proteins. Accumulating evidence has shown that a relevant fraction of lncRNAs show a striking cancer-enriched expression pattern, suggesting an important role in tumor biology [24,25]. These mechanisms include epigenetic reprogramming, the upregulation of oncogene expression, and the stabilization of key cancer-related proteins, and they have led to the promotion of multiple oncogenic mechanisms and the acquisition of therapeutic resistance [25,26]. The large diversity of functions, along with the huge number of different lncRNAs so far identified, presents many opportunities for lncRNAs to act as oncogenes and tumor suppressors, and reports of such roles have accelerated greatly in the past few years [27]. Since lncRNAs can act at different levels, they have been considered suitable as biomarkers for early cancer detection and anticancer treatment monitoring, as well as therapeutic targets. CircRNAs have recently emerged as non-coding, regulatory RNAs expressed in all cells and tissues, being single cell able to express thousands of circRNAs. They are covalently closed transcripts formed through an RNA back-splicing event and have a very long half-life. CircRNAs have the potential to act as oncogenes or tumor suppressors, and alterations in the gene copy number or transcription of circRNA precursors change their levels in some cancers. Hundreds of thousands of different circRNAs have been detected by deep sequencing of RNA from patient tumors and cancer cell lines, and some of them were found to be overexpressed in cancers compared with the respective normal tissues, which raises the possibility of their use as biomarkers of disease [27,28]. 

Mechanistically, ncRNAs are involved in gene regulation at several levels, from epigenetic gene silencing to the post-transcriptional regulation of mRNA balance, including chromatin remodeling and signal transduction [29]. NcRNAs can circulate in biological fluids as free circulating ncRNA, packaged into extracellular vesicles, such as microvesicles and exosomes, or bound to ribonucleoprotein complexes, such as RNA-binding proteins and lipoproteins. Extracellular vesicles and ribonucleoprotein complexes protect ncRNAs by ribonucleases and make them stable in biofluids [30]. Given their ease of access, abundance, and stability, circulating ncRNAs could offer an appropriate answer to the present gap of reliable biomarkers for the diagnosis and prognosis of RCC. In this review, we analyze the possible role of circulating ncRNAs as new promising diagnostic and prognostic biomarkers of RCC in day-to-day clinical practice using a critical approach with regard to the study design and to the main preanalytical and analytical confounders in circulating ncRNA detection. 

## 2. MiRNAs in Plasma/Serum Samples

Most studies have reported serum or plasma miRNAs as potential novel and non-invasive screening, diagnostic, or prognostic biomarkers for RCC patients [31,32,33,34,35,36,37,38,39,40,41,42,43,44,45,46,47,48,49,50,51,52,53,54,55,56,57,58,59]. These miRNAs could lack a high sensitivity or specificity if detecting alone, but a combination of miRNAs, a “miRNA signature”, either alone or in combination with the traditional clinical-pathological features, could show a high discriminatory power [31,32,34,35,36,38,39,41,42,43,47,52,53,55]. Despite they are promising, many of the potential miRNA biomarkers for RCC need further validation studies. Currently, none of these strategies based on serum/plasma circulating miRNAs have entered clinical practice yet. Available studies concerning plasma/serum miRNAs often showed inconsistent results and were difficult to compare with each other due to the different study designs and pre-analytical and analytical conditions [31,32,33,39,41,43,47,52]. In most studies, serum was used as the source of RNA, even if serum samples could present an altered miRNA composition due to the release of extracellular vesicles containing miRNAs from platelets during the coagulation process [60]. Consequently, plasma samples could be considered the blood component of choice for circulating miRNA analysis. In addition, very few studies evaluated sample haemolysis [38,52], even if the haemolysis of blood samples could alter circulating miRNA levels by up to 50-fold, and an erythrocyte contamination of only 0.008% could impair serum/plasma miRNA quantification [61]. Moreover, the information on (i) the collection tubes used, (ii) the processing time from blood collection to plasma/serum preparation, and (iii) the centrifugation speeds, times, and temperature used to separate plasma/serum from whole blood were often missing or differed among different studies (Table 1).

In addition, different RNA extraction methods have been used, as well as different reagents and qPCR instrument platforms for the amplification and detection of miRNAs (Table 1). Some protocols include a pre-amplification step before to carry on qPCR in order to enhance the input amount of low-abundant miRNAs, which can be, in biological fluids, very low even for conventional qPCR [32,34,41,43,46]. Furthermore, different data normalization strategies have been used. MiRNA expression levels were normalized using different endogenous [31,37,40,41,42,48,49,53,55,56] or exogenous controls [33,35,36,39,46,47,50,52], a combination of them [54], or a panel of endogenous controls [34,38,43,44], while in one study, the authors used non-normalized data for their statistical analysis [32]. The determination of suitable reference genes, which should be used for the normalization of circulating miRNA expression, remains a highly controversial issue. On the other hand, the use of non-normalized data prevents the elimination of intrinsic differences between samples or technical variability among samples (such as a variation in the starting material or reaction efficiency during reverse transcription or qPCR), thus severely limiting the accuracy of the study. Moreover, the authors have almost always used a candidate miRNA approach for their investigations [31,32,33,35,36,37,38,39,40,42,44,45,47,48,49,50,52,53,55,56,57,58,59], with consequent limitations in the selection of the most promising miRNAs. To the best of our knowledge, there were only six genome-wide studies performed to date in the serum or plasma of RCC patients [34,41,43,46,51,54]. Among the authors of these six genome-wide studies, only Chanudet et al. (2017) and Lou et al. (2017) used plasma samples for their analysis, while the authors of the other works used serum samples, which, as we highlighted before, had different miRNAs profiles [51,54]. Furthermore, the authors used different global screening methods for the detection and quantification of miRNAs: TaqMan Low Density Arrays technology [41,43,46,51], microarray platforms [54], and small RNA sequencing [34]. The TaqMan Low Density Arrays technology is a real-time PCR-based technology, with a high sensitivity, high specificity, and high dynamic range, while microarrays and small RNA sequencing showed differences in their sensitivity and sensibility in miRNA expression analysis [80,81]. The validation phase, where present, was always performed with RT-qPCR, but again with different reagents and platforms for the amplification and detection of miRNAs and different data normalization strategies (Table 1). One of the major limitations regarding five out of the six genome-wide studies is that the screening was limited to small numbers of samples (5 [54], 6 [46], 15 [41], 18 [34], and 25 [43] serum/plasma samples from RCC patients), thus severely limiting the power of the statistical analysis and increasing the risk of false negative and false positive results. The study by Chanudet et al. (2017), conversely, provided the largest exploratory study of plasma circulating miRNAs of RCC patients reported to date, with a study cohort of 94 cases and 100 controls [51]. Moreover, apart from Chanudet et al. (2017), the authors of the other genome-wide studies used different approaches in the selection of miRNAs for subsequent validation phases, thus further limiting the comparability of the studies. Redova et al. (2012) compared the serum miRNA expression levels between ccRCC patients and matched healthy controls and identified the most promising miRNAs based on the differential expression in terms of the fold change and *p*-value, previous observations, and biological plausibility based on the literature data, and high expression levels [41]. On the other hand, Wulfken et al. (2011) chose as miRNAs to be further validated those presented at the highest levels and upregulated both in the serum and tissue of RCC patients, based on the hypothesis that the upregulation of miRNA expression in RCC tissue should be reflected in higher miRNA serum levels [46]. Wang et al. (2015) used a different approach and identified markedly dysregulated miRNAs using two pooled samples, instead of individual samples, from low-stage RCC patients and non-cancer controls [43]. Heinemann et al. (2018) compared serum miRNA expression levels in ccRCC patients with benign renal tumors, selecting differentially expressed miRNAs that were strongly expressed in serum and that had not been studied in serum by other researchers [34]. Finally, Lou et al. (2017) chose to compare the miRNA expression levels between paired pre-operative plasmas and post-operative 7-day plasmas of ccRCC patients [54]. Chanudet et al. (2017) were the only ones to perform a large-scale analysis in a study cohort of almost 200 patients comprising ccRCC patients and controls, without a validation phase but including an internal bootstrapping validation approach [51]. Consequently, they finally found different miRNAs with potential diagnostic or prognostic utility for RCC. Serum miR-1233, identified as the most promising in the discovery and verification cohorts by Wulfken et al. (2011), showed a poor diagnostic power in the multicenter validation cohort. Indeed, serum miR-1233 was able to discriminate RCC patients (*n* = 84) from healthy individuals (*n* = 93) with an AUC value of only 0.588 (95% CI 0.505–0.671) and a sensitivity and a specificity of 77.4% and 37.6%, respectively [46]. In addition, serum miR-1233 was not correlated with clinical-pathological parameters and was present in a similar level in patients with angiomyolipoma (*n* = 3) or oncocytoma (*n* = 10) and RCC patients [46]. However, two subsequent independent studies found miR-1233 upregulated in both the serum and plasma of RCC patients, compared to healthy controls [47,53]. In these studies, serum miR-1233 was able to diagnose ccRCC patients with a sensitivity of 93.3% and a specificity of 100% [47], while the plasma levels of miR-1233 showed a prognostic potential [53], with an addictive effect when combined with other miRNAs. Consequently, further studies are needed to evaluate the clinical utility of serum/plasma miR-1233 in RCC diagnosis or prognosis. Conversely, Lou et al. (2017) validated miR-144-3p as a very promising diagnostic biomarker for RCC [54]. Plasma miR-144-3p was over-expressed in ccRCC patients (*n* = 106) and could successfully discriminate ccRCC patients from both healthy individuals (*n* = 123) and angiomiolipoma patients (n = 28), yielding an AUC of 0.91 (95% CI, 0.88–0.95; sensitivity = 87.1%; specificity = 83.02%) and 0.82 (95% CI, 0.74–0.91; sensitivity = 75%; specificity = 71.7%), respectively [54]. Plasma miR-144-3p could also have a role as a follow-up biomarker of ccRCC. Indeed, it showed a significant post-operative decrease and was found to be higher than the post-operative levels in 2 patients that developed recurrence post-operatively [54]. Moreover, higher levels of miR-144-3p were positively correlated to tumor tissue in ccRCC patients and were associated with advanced pT stages, suggesting a role of miR-144-3p as an oncomiR involved in ccRCC development [54]. However, a multicenter study is required to further confirm the results obtained by Lou et al. (2017) in order to use plasma miR-144-3p in the clinical management of ccRCC patients. Instead of a single biomarker, Redova et al. (2012) and Wang et al. (2015) found a RCC diagnostic panel of two and five miRNAs, respectively [41,43]. The panel comprising serum miR-378, which was upregulated in RCC patients, and miR-451, which was downregulated in RCC patients, had an AUC of 0.86 and a sensitivity and a specificity of 81% and 83%, respectively, in discriminating cases (*n* = 90) from controls (*n* = 35) [41]. Taken alone, the same miRNAs had a lower discriminating power [41]. The panel of five serum miRNAs, including miR-193a-3p, miR-362, and miR-572, which were upregulated, and miR-28-5p and miR-378, which were downregulated in RCC patients (*n* = 107), compared to the non-cancer controls (*n* = 107), seems very promising for clinical use, with an AUC of 0.796 (95% CI, 0.724–0.867) [43]. Furthermore, the five miRNAs panel was able to differentiate patients with ccRCC in the early stages of the disease from healthy controls with an AUC of 0.807 (95% CI, 0.731–0.871) and a sensitivity and a specificity of 80% and 71%, respectively [43]. However, there were ambiguous results concerning serum miR-378. Higher serum miR-378 levels in RCC patients with respect to healthy controls were found in other two studies by Fedorko et al. (2015) and Hauser et al. (2012), which is in agreement with Redova et al. (2012) and in contrast to Wang et al. (2015) [32,33,41,43]. Fedorko et al. (2015) showed that serum miR-378 was able to discriminate RCC patients (*n* = 195) from healthy controls (*n* = 100) with an AUC of 0.82 [32]. In addition, an increased level of serum miR-378 was positively correlated with disease-free survival (*p* = 0.036) and clinical stage (*p* = 0.0476), but serum miR-378 could not be considered as an independent prognostic factor in RCC, as shown by multivariate analysis [32]. On the other hand, Hauser et al. (2012), while showing that serum miR-378 was upregulated in RCC patients in a discovery cohort (25 ccRCC patients vs. 25 controls) and discriminated cancer patients form healthy controls, they could not confirm their results in the validation cohort (117 RCC patients vs. 123 control subjects) [33]. The inconclusive results regarding serum miR-378 questioned its clinical utility in RCC diagnostics. In the last two genome-wide studies, Heinemann et al. (2018) and Chanudet et al. (2017), while not finding serum/plasma miRNAs to have diagnostic potential, identified miRNAs with prognostic utility for ccRCC [34,51]. Heinemann et al. (2018) showed that high serum miR-122-5p and miR-206 levels were associated with adverse clinical-pathological parameters, as well as with a shorter period of progression-free, cancer-specific, and overall survival in patients with ccRCC [34]. Chanudet et al. (2017) found that lower plasma circulating levels of miR-150 were significantly associated with both overall and ccRCC-specific survival, even when adjusting for age, sex, and conventional staging, which marginally improved their predictive accuracy (bootstrap optimism-corrected AUC of 0.81 versus 0.78) [51].

As reported before, apart from those six genome-wide studies, in all the other studies on serum/plasma free circulating miRNAs, the authors have used a candidate miRNA approach for their investigations, and only four studies have included a validation phase [35,36,39,44]. Several studies found increased serum and plasma miR-210 levels in RCC patients [32,37,40,49,52,53], and higher miR-210 levels were also found in serum-derived EVs of RCC patients, which is better explained later on in this review [62,63]. The sensitivity, specificity, and area under curve (AUC) for the miR-210 levels determined using Receiver Operator Characteristic (ROC) analysis varied among the different studies due, as we highlighted before, to the different study designs and pre-analytical and analytical conditions. Most of all, the expression level of miR-210 was normalized using different endogenous or exogenous controls (Table 1), while Fedorko et al. (2015) was the only one to include a pre-amplification step and non-normalized data for their statistical analysis [32]. In addition, the sample size was different among these studies, ranging from 32–34 RCC patients and 23 healthy controls [37,40] to 195 RCC patients and 100 healthy controls [32]. Moreover, Fedorko et al. (2015) analyzed patients with an RCC of various subtypes, Liu at al. (2016) did not specify the tumor histology of RCC, while all the other authors only analyzed samples from ccRCC patients. Despite all these differences, the results obtained in these studies highlighted the diagnostic potential of serum miR-210 [32,37,40,49], while Dias et al. (2017) demonstrated the prognostic potential of plasma miR-210 in RCC patients [53]. Zhao et al. (2013) showed that miR-210 was able to clearly distinguish ccRCC patients and healthy controls, with an AUC of 0.874, a sensitivity of 81.0%, and a specificity of 79.4% [49]. Iwamoto et al. (2014), in a study cohort comprising mostly ccRCC in the early stage (85.3% of all cases), obtained an AUC of 0.77 (95% CI, 0.65–0.89) and a sensitivity and specificity of 65% and 83%, respectively [37]. Liu et al. (2016) did not perform Receiver Operator Characteristic (ROC) analysis, but differences in the serum level of miR-210 between RCC patients and healthy controls were compared using a t-test, and the results are statistically significant (*p* < 0.001) [40]. In the remaining studies, miR-210 was not considered alone, but rather in combination with other miRNAs [32,52,53]. Fedorko et al. (2015), as explained before, while demonstrating the diagnostic potential of serum miR-210, showed that a high serum level of miR-378 was better able to discriminate RCC patients from controls than miR-210, with AUCs of 0.74 and 0.82 for miR-210 and miR-378, respectively. Nonetheless, miR-210 and miR-378 combined had a higher discriminating power, with an AUC of 0.848 and sensitivity and specificity of 80% and 78%, respectively [32]. The study by Dias et al. (2017), already mentioned above, showed that a panel of three plasma miRNAs, including miR-210, miR-221, and miR-1233, upregulated in RCC patients (*n* = 54) with respect to controls (*n* = 50), had a prognostic potential for RCC. Indeed, combined higher levels of these three miRNAs were significantly associated with a higher risk of specific death by RCC (HR = 3.02, 95% CI 1.19–7.64, *p* = 0.014) [53]. Moreover, this miRNA panel improved the predictive ability of standard prognostic variables used in the clinic, such as tumor stage, Fuhrman grade, age, and gender [53]. Chen et al. (2018), on the other hand, demonstrated that plasma miR-210, miR-224, and miR^210 × 224^ had a good sensitivity but poor specificity and relatively low accuracy in distinguishing ccRCC patients from healthy individuals, even if the expression levels of plasma miR-210 and miR-224 were significantly increased in ccRCC patients (*n* = 66), compared with healthy controls (*n* = 67) [52]. In addition, Kalogirou et al. (2020) showed that miR-210-3p, alone or in combination with miR-21-5p, had a poor diagnostic utility for papillary RCC, while it seemed to have prognostic utility [38]. However, there was strong evidence for a potential clinical utility of serum/plasma miR-210 as an RCC biomarker. Corroborating this hypothesis, miR-210 was significantly upregulated in primary RCC tissues, compared with normal tissue [40,49], matching its circulating levels in the blood of RCC patients. Moreover, plasma miR-210 was decreased significantly post-operative [32,52,59,63], suggesting that the serum miR-210 expression level was closely related with kidney cancer tissue and could be used also as follow-up biomarker. Liu et al. (2016) also demonstrated that human renal carcinoma ACHN cell proliferation and invasion were significantly increased, and apoptosis was significantly decreased, when miR-210 was overexpressed and vice-versa when miR-210 was inhibited [40].

Some other miRNAs, in addition to miR-378, miR-1233, and miR-210, previously discussed, were reported to be deregulated in the plasma or serum of RCC patients in at least two independent studies, corroborating or contradicting previous results [31,36,39,42,47,52,57]. The authors of these works found different miRNA panels of potential clinical utility as diagnostic or prognostic biomarkers of RCC, with some overlaps in the composition of miRNA panels, but not always with consistent results.

In the study by Cheng et al. (2013), miR-141 was downregulated, and miR-224, miR-21, and miR-34a were upregulated in the sera of patients with ccRCC (*p* < 0.01), compared with those of patients with benign kidney lesions, which is consistent with their expression in paired tumor tissue samples [31]. On the other hand, a miRNA panel comprising miR-34a and miR-141, which was downregulated, and miR-1233, which was upregulated in ccRCC patients (*n* = 30) with respect to controls (*n* = 15), was able to diagnose ccRCC with a 100% sensitivity and 60% specificity, while the combination of only serum miR-141 and miR-1233 was the most powerful, showing a 100% sensitivity and 73% specificity, as reported above in this review [47]. The expression levels of these miRNAs matched that of tumor tissue with respect to adjacent normal tissues [47]. Another two miRNAs, miR-21 and miR-106a, both upregulated in the serum of ccRCC patients (*n* = 30) with respect to that of healthy controls (*n* = 30) (*p* < 0.0001), could diagnose ccRCC with an AUC of 0.865 (95% CI: 0.766–0.965; sensitivity = 77.3% and specificity = 96.4%) and 0.819 (95% CI: 0.710–0.929; sensitivity = 86.7% and specificity = 70.0%), respectively [42]. Notably, miR-21 and miR-106a serum levels were significantly decreased a month after surgery, compared with the pre-operative samples (*p* < 0.0001), while there was no statistically significant difference between the post-operative and healthy controls serum samples, suggesting that serum miR-21 and miR-106a could have arisen from the primary renal tumors [42]. Another miRNA panel, discovered in a total cohort of 126 RCC patients and 130 controls divided into screening (two pooled samples of RCC (*n* = 20) and controls (*n* = 20)), testing (RCC = 30, controls = 30), and validation sets (RCC = 76; controls = 80), included miR-224-5p, miR-34b-3p, and miR-182-5p [36]. MiR-224-5p was upregulated, while miR-34b-3p and miR-182-5p were downregulated in the serum of RCC patients, compared with controls, and had the highest potency among all analyzed miRNAs (*n* = 30) selected from the literature on the differentiation of RCC from controls [36]. The combination of these three miRNAs had a higher diagnostic ability than individual miRNA, with an AUC of 0.879 (95% CI, 0.795–0.963; sensitivity = 80.0%, specificity = 69.0%) in the testing set and 0.855 (95% CI, 0.797–0.912; sensitivity = 80.3%, specificity = 66.3%) in the validation set. Another promising diagnostic panel comprised serum miR-508-3p (previously discussed), which was significantly downregulated, and serum miR-885-5p, which was significantly upregulated, in ccRCC patients, compared with healthy controls [39]. This miRNA panel, discovered in a small cohort of 10 ccRCC patients and 10 healthy controls, in the validation cohort could diagnose ccRCC with an AUC value of 0.90 (95% CI, 0.84–0.96) [39]. The serum miR-508-3p expression was significantly correlated with the T stage (*p* = 0.004), metastasis (*p* = 0.009), Fuhrman grading (*p* < 0.001), and TNM stage (*p* = 0.005), while the serum miR-885-5p expression was significantly correlated with the T stage (*p* < 0.001) and Fuhrman grading (*p* = 0.013). Kalogirou C. et al. (2020), already mentioned above in this review, were the first to investigate serum circulating miRNAs in patients with papillary RCC (pRCC) in relation to their potential ability to discriminate pRCC from healthy controls (*n* = 33) and pRCC type 1 (*n* = 34) from type 2 (*n* = 33) in a multicenter study [38]. Previous analyses were limited to a few patients belonging to larger cohorts comprising patients with all histological subtypes. They showed that the serum expression levels of 11 miRNAs, selected from the Cancer Genome Atlas (TCGA) pRCC study, because they were highly and differentially expressed, did not significantly discriminate healthy individuals from patients with pRCC, nor patients with type 1 from those with type 2 pRCC [38]. However, miR-21-5p serum levels were significantly increased in patients with advanced pRCC (>pT3, and/or pN+ and/or pM+) in comparison to those with localized pRCC or in control subjects [38]. In addition, serum miR-210-3p levels were significantly lower in patients with localized tumors in comparison to healthy controls [38]. However, both miRNAs, even in combination, showed a very low diagnostic ability, and their potential as prognostic tool for pRCC has to be validated [38]. Consequently, the need to identify deregulated miRNAs by global screening approaches and to validate them in larger patient cohorts in order to find clinically relevant biomarkers for pRCC was very clear. Finally, Teixeira A.L. et al. (2014) demonstrated that circulating plasma miR-221 and miR-222 had a significantly increased expression in RCC patients (*n* = 43), compared with controls (*n* = 34). However, the discriminating ability of miR-221 could not be of diagnostic utility, presenting an AUC of 0.696 (95% CI: 0.499–0.893), with a sensitivity of 72.5% and a very low specificity of 33.3%. Nevertheless, higher circulating expression levels of miR-221 were associated with clinical metastasis and microvascular invasion [56]. Consistently, high expression levels of miR-221 were associated with a lower overall survival (48 vs. 116 months; *p* = 0.024) and lower cancer-specific survival (HR = 10.7; 95% CI: 1.33–85.65; *p* = 0.026) in multivariate analysis, using the TNM stage, Fuhrman nuclear grade, and age (≥60 years) as covariates [56]. Furthermore, the addition of circulating plasma miR-221 expression information to the tumor characteristics (tumor TNM stage and Fuhrman nuclear grade) and age (≥60 years) had a higher capacity to predict the risk of death by RCC (increasing c index from 0.800 to 0.961), suggesting that plasma miR-221 could be a useful tool as a new molecular independent prognosis factor in RCC [56]. In a subsequent work, Teixeira A.L. et al. (2015) demonstrated that the combined effect of EGF + 61G > A and TGFB1 + 869T > C polymorphisms, associated with higher levels of EGF and lower TGFβ1 production, finally had an unfavorable synergic effect on the progression-free interval and overall survival of RCC patients [55]. In RCC patients (*n* = 22), plasma miR-7, miR-221, and miR-222 were upregulated, compared with healthy individuals (*n* = 27), which is consistent with their previous work [55]. Moreover, the patients carrying those polymorphisms, defined as intermediate/high genetic proliferation profile carriers, presented an increase in the expression levels of these miRNAs during the RCC development, while low genetic proliferation profile carriers did not. Consequently, miR-7, miR-221, and miR-222 plasma levels could also be useful phenotype biomarkers of EGFR/MAPK activation [55].

Other serum/plasma miRNAs may be of potential diagnostic or prognostic clinical interest, as shown by other 7 studies [35,44,45,48,50,58,59]. Serum miR-200a could be a potential non-invasive diagnostic biomarker for early-stage renal cell carcinoma [44]. Indeed, Wang C. et al. (2019) showed that miR-200a was consistently decreased in RCC patients’ serum in a discovery cohort of 26 RCC patients and 26 non-cancer controls, and this result was validated in a validation cohort of 73 patients and 73 controls [44]. Serum miR-200a was able to differentiate RCC patients from normal controls with an AUC of 0.836 (95% CI, 0.728–0.944) for the training phase, 0.702 (95% CI, 0.618–0.785) for the validation phases, and 0.724 (95% CI, 0.655–0.793) for the combined two phases [44]. Serum miR-200a was also able to discern stage I RCC cases, stage II RCC cases, and stage I–II RCC cases from controls with AUCs of 0.740 (95% CI, 0.667–0.814), 0.700 (95% CI, 0.544–0.847), and 0.733 (95% CI, 0.662–0.804), respectively [44]. Urinary miR-200a also showed potential clinical utility [44]. In addition, a three-miRNA panel with diagnostic ability, comprising miR-20b-5p, miR-30a-5p, and 196a-5p, was found by Huang et al. (2020) in a cohort of 110 RCC patients and 110 healthy volunteers, divided into testing and validation sets [35]. This miRNA-panel was able to discriminate cases from controls with an AUC of 0.949 (95% CI, 0.918–0.980; sensitivity = 92.8%, specificity = 80.0%) in the testing set and 0.938 (95% CI, 0.889–0.988; sensitivity = 92.5%, specificity = 80.0%) in the validation set, while, taken alone, these miRNAs had a lower discriminating power [35]. Another miRNA that could be used as a non-invasive diagnostic biomarker for ccRCC is serum miR-625-3p [50]. Serum miR-625-3p was significantly downregulated in ccRCC patients (*n* = 50), compared with healthy individuals (*n* = 74) (*p* < 0.001). Conversely, miR-625-3p was upregulated in ccRCC tissues, compared with paired normal renal tissues, suggesting that miR-625-3p retained in tumor tissues might contribute to the ccRCC malignant phenotype [50]. This serum miRNA had a good discriminatory power, with an AUC of 0.792 (95% CI, 0.714–0.870, sensitivity = 70.3% and specificity = 80.0%) [50]. Serum miR-429 showed, instead, potential as both a diagnostic and prognostic biomarker for RCC [45]. Indeed, the serum miR-429 level in RCC patients (*n* = 27) was higher than in non-cancer patients (*n* = 28), and patients expressing lower miR-429 serum levels showed better clinical outcomes after a conventional treatment [45]. Furthermore, plasma miR-187, which was significantly downregulated in ccRCC patients, compared with normal individuals [59], and plasma miR-483-5p, which was significantly upregulated in ccRCC patients (*n* = 12) after nephrectomy [58], could be of diagnostic clinical utility, but further in-depth studies are still needed. Finally, Zhang Q. et al. (2015) showed, in a cohort of 82 RCC patients and 19 healthy volunteers, that miR-183 was significantly upregulated in the serum of RCC patients and that its level was positively associated with the grading of RCC [48]. They also demonstrated that primary RCC cells from patients with high serum miR-183 were less sensitive to the cytotoxicity induced by NK cells. Therefore, serum miR-183 could be a useful predictive biomarker of the response of RCC cells to the cytotoxicity induced by NK cells. Targeting miR-183 could also be a new therapeutic strategy for improving the outcome of NK cell-based immunotherapy [48].

## 3. Long Non-Coding RNA in Serum

In clinical practice, the assessment of oncogenic lncRNAs in biofluids represents an opportunity for early cancer detection and monitoring. Furthermore, as predictors of sensitivity to anti-cancer treatments, lncRNAs could be integrated into precision medicine strategies [26]. However, although the utility of circulating lncRNAs as biomarkers in cancer and possible pathological mechanisms have been repeatedly reported in RCC, the data remain limited. In addition, most lncRNAs has been investigated as biomarkers in tumor tissue and compared to the adjacent non-cancerous part of the kidney, while so far, few studies include the assessment of lncRNA in biofluids. A panel of 82 cancer-associated lncRNAs were assessed in serum samples collected from 71 ccRCC patients, 62 age- and sex-matched healthy controls, and 8 patients with benign renal tumors, and a 5-lncRNA signature, including lncRNA-LET, PVT1, PANDAR, PTENP1, and linc00963, was identified [82]. The panel was validated in a training set of 24 ccRCC patients and 27 normal controls and in a testing set of 37 ccRCC patients and 35 healthy controls, successfully discriminating non-cancer from RCC patients (AUC = 0.900 and 0.823 respectively). The predictor retained their diagnostic ability throughout the different clinical TNM stages, with an AUC of 0.85 for stage I tumors and 0.80 for stages II–IV tumors. In an additional cohort (10 ccRCC and 8 benign renal tumor subjects), the cancer patients showed significantly higher risk indices, as compared to controls [82]. The gradual increase of lncRNA during hepatocarcinogenesis (GIHCG) was first described in hepatocellular carcinoma, and its oncogenic role has been reported in various cancers, including RCC [83]. The levels of GIHCG were found to be significantly upregulated in the sera of 46 RCC patients and 46 matched healthy controls, which is correlated with an increase in RCC tissues, compared with adjacent normal renal tissues and with advanced TNM stages. The GIHCG serum levels were able to discriminate RCC patients and age- and sex-matched healthy individuals with an 87.0% sensitivity and 84.8% specificity (AUC = 0.920). It was also able to discriminate early-stage (TNM stage I) RCC patients from healthy controls with an 80.7% sensitivity and 84.8% specificity (AUC = 0.886). Furthermore, serum GIHCG was significantly reduced after the radical resection of RCC [83]. 

## 4. Urinary miRNAs

Urine provides an alternative to blood serum or plasma as a potential source of tumor biomarkers, since it is a body fluid that is easy to collect with non-invasive procedures, which favors the patients’ compliance. It is not subjected to homeostatic mechanisms, and its contents reflect many changes of the body, such as pregnancy, aging, and disease, especially of the urogenital tract. Most studies utilize total urine samples, a complex mixture of salts, proteins, metabolites, cells, and debris, which are sometimes very diluted. These components mostly originate from the upper and lower urinary tract but may also be filtered directly from the systemic circulation, if they are smaller than 6–8 nm. Therefore, urine is enriched in molecules derived from urinary organs, especially the kidney and bladder, representing a unique advantage in the study of the physiopathology of these organs. However, due to its special physiological role, urine often shows big volume fluctuations in different individuals or at different stages of the same individual, causing a serious issue in terms of normalization and standardization. To identify the best miRNA candidates for urinary biomarkers of ccRCC, expression data on 340 miRNAs from the Gene Expression Omnibus and the European Bioinformatics Institute datasets, generated using miRNA microarray platforms on ccRCC specimens and kidney tissues from healthy subjects, were analyzed [72]. Most performing candidates were tested in ccRCC and adjacent non-cancerous kidney tissue specimens from 14 patients. MiR-122, miR-1271, and miR-15b were found to be potentially interesting markers, and their presence was tested in the urine of 14 healthy subjects and 13 ccRCC patients, defining a score (7p-urinary score) to evaluate the presence of ccRCC in patients (AUC = 0.96, with a 100% sensitivity and 86% specificity) [72]. Let-7 family miRNA levels were investigated in 105 first morning urine specimens collected from 69 non-metastatic ccRCC patients and 36 gender- and age-matched healthy controls [74]. All let-7 miRNAs (let-7a, let-7b, let-7c, let-7d, let-7e, and let-7g) were significantly increased in the urine samples obtained from RCC patients, compared to healthy controls, and let-7a outperformed the others and were able to differentiate between cases and controls (AUC = 0.8307, with a 71% sensitivity and 81% specificity) [74]. Di Meo et al. focused on small renal mass ccRCC and screened, by microarray analysis, 754 miRNA in 80 urine samples, including 30 renal oncocytoma (≤4 cm) cases and 26 progressive and 24 non-progressive small renal mass ccRCC cases [73]. Small renal mass RCC need to be accurately stratified according to risk to avoid unnecessary treatments. Nine urinary miRNAs displaying a significantly elevated expression in small renal mass ccRCC relative to renal oncocytoma (≤4 cm) were identified, and miR-328-3p exhibited a significantly downregulated expression in progressive relative to non-progressive small renal mass ccRCC. Patients with an elevated miR-328-3p expression had a significantly longer overall survival, compared to patients with a low miR-328-3p expression. Since miR-328-3p was not significantly associated with gender, age, laterality, tumor size, or grade, it was suggested to be an independent prognostic biomarker [73].

Since miR-210 has been proven to be overexpressed in ccRCC patients, its presence in urine as a potential tool of liquid biopsy for ccRCC was examined [75]. Urine samples from 75 patients with a ccRCC and 45 control subjects without a cancer were analyzed, and its levels were found to be significantly higher in patients, discriminating them from healthy subjects (AUC = 0.76, with a 57.8% sensitivity and 80.0% specificity). As a confirmation of the tumoral origin, the expression level of urinary miRNA-210 was significantly decreased in the patients a week after surgical intervention [75]. In another study, miR-210-3p levels were analyzed in neoplastic and healthy tissue and in urine specimens collected during surgery and during follow-up of 21 ccRCC patients, confirming that the expression of miR-210-3p was upregulated in both and significantly reduced in urine derived from disease-free patients (from 3 to 12 months). In addition, the urinary levels of miR-210-3p correlated with responsiveness to the therapy in a subset of metastatic ccRCC patients [78]. Analogously, the expression of miR-15a, a tumor suppressor promoting apoptosis and inhibiting cell proliferation by targeting multiple oncogenes, was measured in the urine of 67 patients with solid renal tumors, including ccRCC (*n* = 22), papillary RCC (*n* = 16), chromophobe RCC (*n* = 14), oncocytoma (*n* = 8), papillary adenoma (*n* = 2), and angiomyolipoma (*n* = 5), and compared to 15 healthy volunteers without a kidney pathology [76]. A difference in the mean miR-15a expression was observed in groups of patients with RCC and benign renal tumors and healthy persons. Eight days after nephrectomy, miR-15a decreased by 99.53% in patients with RCC. The urinary miR-15a expression differentiated RCC from benign renal tumors with an AUC = 0.955 (98.1% specificity and 100% sensitivity), indicating that it is a good candidate as a diagnostic molecular marker for RCC [76]. In another study, urines from renal tumor patients, including 10 ccRCC, 5 chromophobe RCC, 6 papillary RCC, 5 benign renal oncocytoma, and 5 healthy controls, were analyzed immediately prior to the operation and one-week post nephrectomy [79]. Four miRNAs, miR-498, miR-183, miR-205, and miR-31, were demonstrated to be increased in oncocytoma samples and proposed as presurgical urinary biomarkers due to their known regulatory mechanism in such tumors. MiR-183 appeared to be the best candidate in the urinary diagnostic approach to identify oncocytomas, and its levels in patients’ urines 1 week after surgery dropped to about 5% of the previous values [79].

Epigenetic regulatory mechanisms, such as genome-wide DNA methylation, are strongly involved in the pathogenesis of human tumors, including ccRCC, and may be used for diagnostic and prognostic purposes [84]. Since miR-30a-5p was proposed as an onco-suppressor in ccRCC, its methylation status in tissues and urines from ccRCC patients was determined [77]. Two significant hypermethylated CpG loci, which are correlated with miR-30a-5p transcriptional downregulation, were disclosed. In urine sediment samples, miR-30a-5p promoter methylation levels identified cancer both in testing (53 ccRCC patients, 57 healthy donors, with AUC = 0.684, an 83% sensitivity, and a 53% specificity), and validation cohorts (171 ccRCC patients and 85 healthy controls, with AUC = 0.67, a 63% sensitivity, and a 67% specificity). Furthermore, higher miR-30a-5p promoter methylation levels independently predicted metastatic dissemination and survival [77].

## 5. Extracellular Vesicles

By resembling the tumor cells of origin, EVs can be considered as a more accessible source of multiple biomarkers. They have been found in body fluids, including blood and urine, which are among the most studied liquid biopsies. Their isolation and analysis are quite challenging because of their small sizes (hundreds of nanometers or even less) and low densities. Many systems and new techniques have been developed for EV recovery from different biological fluids, aiming at making the yield consistent with the specificity. Ultracentrifugation, precipitation, size-exclusion chromatography, and immune-affinity purification are among the most employed systems, which exploit EV size, density, and composition. However, each biological fluid presents specific physical and biochemical characteristics, thus requiring ad hoc isolation and purification procedures. Moreover, the development of an appropriate workflow is dependent on the availability of the starting material, the required purity grade of isolated EVs, and the available equipment, all of which have to be adapted to the clinical settings to promptly and effectively translate scientific discoveries into public health actions. A key point is the routine biobanking of EVs, which needs optimized and standardized isolation and storage protocols to guarantee feasible collection and reproducible results [85].

Being surrounded by a double lipid layer, EVs protect their content from the environment, improving half-life and stability. RNAs can be actively loaded into EVs by cancer cells in order to support cancer development and spread [86]. Thus, EVs represent an enriched source of biomarkers, compared to the whole fluid where they are floating. In addition, they are expressed on the surface multiple tumor markers, which can be exploited to isolate them by affinity purification and select them among all the other EV populations in the fluid.

Studies on the specific cargo analysis of extracellular vesicles in RCC are mainly focused on miRNAs, which are often selected on the basis of data derived from the miRNA expression in tumor tissues. In a retrospective study, the expression levels of EpCAM-positive EV miR-210 and miR-1233 were found to be significantly higher in sera from 82 ccRCC patients than in 80 healthy individuals (AUC of 0.69, with a 70% sensitivity and 62.2% specificity for miR-210, and AUC of 0.82, with an 81% sensitivity and 76% specificity for miR-1233) [62]. The expression levels of both EV miRNAs were significantly lower 7 days after the surgical tumor removal than in preoperative samples. In a different study, based on a microarray screening, serum miR-210 was the most upregulated miRNA in 45 patients with ccRCC with respect to 30 healthy controls (AUC = 0.8779, 67.5% sensitivity and 70.0% specificity) [63]. EV miR-210 was confirmed to be significantly higher in cancer patients (AUC = 0.8779, 82.5% sensitivity and 80.0%, specificity), indicating a better performance as a diagnostic biomarker, compared to serum miR-210. EV miR-210 expression levels tended to be higher in patients with T3/T4 tumor stage and positively correlated with metastatic status. EV miR-210 was downregulated gradually at 7 days after surgical intervention, and it showed a two-fold decrease or greater in most RCC patients after 1 month and remained stable after 3 months [63]. Recently, an EV miRNA analysis of plasma samples obtained from RCC patients (5 for the discovery phase by RNA sequencing and 22 for the validation) and controls (5 and 16 subjects respectively) was performed, and a small group of differentially expressed miRNAs was evaluated as a biomarker of RCC [64]. The expression levels of miR-149-3p (AUC = 0.7188, with a 75.0% specificity and 72.7% sensitivity) and miR-424-3p (AUC = 0.7727, with a 75.0% specificity and 81.8% sensitivity) were upregulated, while those of miR-92a-1-5p (AUC = 0.8324, with a 87.5% specificity and 77.3% sensitivity) were significantly downregulated.

EV miRNAs as singles or grouped in a small panel were investigated as prognostic factors as well. For instance, the levels of miR-224 in EVs isolated from the sera of 108 ccRCC patients was correlated to their survival, showing that the high expression group had a significantly shorter progression-free survival, cancer-specific survival, and overall survival, compared with the low-level expression group, indicating that it is an independent prognostic marker [65]. The content of miRNAs in the plasma EVs from two different cohorts (*n* = 44 and 65) of metastatic RCC patients was determined by RNA sequencing and correlated to the overall survival (OS) [66]. High levels of miR-let-7i-5p, miR-26a-1-3p, and miR-615-3p were found to be correlated to an increased OS at 30-month follow up. The mortality rate for patients with a lower expression was 70–80%, while the highest expression of the three miRNAs was associated with a mortality rate of less than 40% [66]. The analysis of a plasma EV 9-miRNAs related to hypoxia and metabolism regulation was carried out in 32 patients with localized ccRCC and 37 with metastatic disease. The levels of EV-derived miR-25-3p, miR-126-5p, miR-200c-3p, and miR-301a-3p were decreased after surgery, whereas miR-1293 EV-levels were increased. Furthermore, metastatic patients presented higher levels of miR-301a-3p and lower levels of miR-1293 when compared to patients with localized disease after surgery [67]. An in silico study assessed the immune-related lncRNA associated with ccRCC survival by integrating three datasets, the Gene Expression Omnibus (GEO), the Cancer Genome Atlas (TCGA), and the Indian Cancer Genome Atlas (ICGA) [87]. A 12-lncRNAs prognostic and independent signature, including AC005104.1, AC093278.2, AC098484.1, AL360181.2, EMX2OS, LINC01011, SPINT1-AS1, AP001372.2, AC007637.1, AL354733.3, AP001189.3, and LINC00886, was identified, since they displayed the best survival prediction, with an AUC of 0.892, 0.790, and 0.792 for a 1-, 3-, and 5-year follow-up in the training dataset, which was confirmed in the testing dataset, with an AUC of 0.587, 0.654, and 0.705, respectively [87].

Not only have serum/plasma EVs been studied, but urine can also be considered as another optimal source of EV biomarkers, particularly for urological malignancies, due to the direct contact of this fluid with the tumor itself [88]. Butz et al. selected 48 miRNAs from their previous work and literature data and tested their expression in RNA isolated from the whole urine or urinary EVs from 28 ccRCC patients and 18 healthy participants by a custom-made miRNA array [68]. In the cell-free miRNAs, only miR-150-5p was significantly overexpressed in ccRCC, with an AUC of 0.66, while EV miR-126-3p was significantly downregulated and able to discriminate between the ccRCC and control groups (AUC: 0.74). The results were validated in an independent cohort of 81 ccRCC patients, 24 patients with benign kidney tumors, and 33 healthy participants: miR-126-3p showed a similar pattern of under-expression in ccRCC patients, with a significant discriminatory power (AUC: 0.65) with respect to healthy controls. In addition, several different combinations of EV miRNAs (including miR-126-3p, miR-486-5p, and miR-34b-5p) could discriminate not only patients with ccRCC, but also those with small renal masses from healthy participants, as well as benign tumors from ccRCC [68]. Differentially expressed miRNAs from urinary EVs were identified using next-generation sequencing and verified using urine samples of 70 early-stage (T1aN0M0) ccRCC patients and 30 healthy donors [69]. The miR-30c-5p expression pattern was significantly lower in ccRCC patients than in control individuals (AUC = 0.8192, with a 68.57% sensitivity and 100%, specificity) but not compared with that of other urinary tumors, such as early-stage (T1N0M0) prostate and bladder cancer patients. In another study, urinary EVs RNA from 6 RCC patients and 6 healthy volunteers were analyzed by sequencing. Among the differentially expressed miRNAs, miR-224-5p was significantly upregulated not only in urinary EVs, but also in cancer tissues, compared to paired adjacent tissues from the same RCC patients [70]. Notably, miR-224-5p was demonstrated to upregulate PD-L1 expression through the cyclin D1/SPOP pathway and to promote the resistance of RCC cells to T cell-dependent toxicity.

Circular RNAs (circRNAs) are a new group of non-coding RNAs that form a continuous loop and are more stable, compared with their linear counterparts. They were detected in EVs as well, and their role has been investigated in the pathogenesis of RCC [71]. EVs were isolated from kidney tissue and plasma specimens obtained from RCC patients (n = 28), and the expression of ~ 5000 genes was evaluated by microarray. Circ_400068 was upregulated in the plasma EVs of patients with RCC and tumor tissues, compared with adjacent healthy controls. Notably, the treatment with circ_400068 containing EVs promoted the proliferation and inhibited the apoptosis of kidney cells. Its activity was suggested to be exerted by targeting the miR-210-5p/SOCS1 axis [71].

## 6. Cohort Selection and Study Population

There are several critical issues related to the study cohort design that should be taken into consideration before conducting a clinical biomarker study. One of the most important hallmarks is related to the sample size determination, which should be decided using a robust power analysis to avoid misleading messages. As a matter of fact, only with an appropriate study cohort is it possible to establish if a different expression in a biological fluid of a ncRNA between two group of patients is statistically significant and related to a real biological difference. Unfortunately, the use of power analysis remains hidden or not well established in some of the analyzed articles, resulting in very small study cohorts. As a consequence, these works can be considered only as descriptive preliminary reports that require further confirmations to enter clinical practice [31,37,40,45,47,58,64,71].

To avoid the issue of an exiguous cohort, considering that RCC is not as prominent as other types of cancers, a few authors decided to create robust multicenter trials to demonstrate their molecular targets in liquid biopsies [32,33,51,77].

The advantage of these works with respect to their monocentric counterpart is related not only to the bigger sample size, but also to the reproducibility of the test derived from specimens of patients enrolled in different institutions and with diverse clinical and laboratory teams.

Another crucial aspect related to the study design is the presence of both discovery than validation cohorts. In fact, different lines of evidence highlighted that the measurement of circulating ncRNAs levels may be affected by a wide variety of both biological and technical variability and required very standardized protocols. For all these reasons, the creation of a validation cohort after the discovery analysis is crucial for generating robust results and reinforcing the reproducibility of the outcomes [33,34,35,36,39,41,43,44,46,66,68,77].

As described in the introduction, RCC comprehends an heterogenous scenario of several renal histologies, along with different stages and grades of aggressiveness. Therefore, each article aimed at detecting new molecular biomarkers in the RCC panorama should provide a detailed description of the oncological clinical variables of the enrolled patients. The majority of the analyzed articles considered all these clinical aspects. However, not all the papers described, at the same time, the different RCC histologies, the TNM classification, and the ISUP WHO grade [45,48,58,59,68,72]. Moreover, some of them did not report the coexisting comorbidities, such as cardiovascular diseases, blood hypertension, metabolic syndromes, renal dysfunction (especially in the urine extraction), systemic autoimmune inflammations or infections, neurological disorders, and concomitant history of other types of tumors (it is remarkable to underline that, for example, chronic systemic inflammations, as mentioned above, could alter the expression of ncRNAs, so multi-variate analysis should always be performed to silence the biological impact of the non-cancerous diseases) [43,47,51]. Last, but not least, an important cornerstone in RCC molecular studies is represented by the definition of the control cohort. First of all, not all the considered works enrolled a control study population, with a ratio of 1.1 or 1.2 with the RCC one. Secondly, because RCC remains one of the most silent and non-specific tumors, the unique strategy to define a patient as a “healthy volunteer” passes through a radiological imaging examination, preferably using a CT scan or an MRI, instead of ultrasound. The authors understand that second-level radiological examinations are not always feasible, and it is not always acceptable to ethical committees to define a patient as a “healthy control” for scientific purposes. However, if the aim of a biological study is the correlation of circulating ncRNA with the presence of RCC, it is essential to avoid possible selection biases derived from “healthy controls” with no history of previous cancer, but with hidden and asymptomatic small renal masses not yet detected [42].

Finally, because of the previous arguments related to the frailty of liquid biopsies, the authors do not consider reliable the majority of the retrospective studies where the biological samples were collected for different purposes and without pre-determined protocols. 

## 7. Conclusions

Renal cancer remains one of the most silent and unexpected tumors, thanks to two different but inextricable aspects: the ability to mimic other pathological conditions, together with the absence of a robust diagnostic molecular tool. The aim of our review was to underline that even though circulating ncRNAs could be promising biomarkers in the diagnosis and prognosis of RCC, further in-depth studies are still needed to allow for their use in clinical practice.

## Figures and Tables

**Table 1:** Circulating ncRNAs as diagnostic or prognostic biomarkers in renal cell carcinoma.

Reference	Biomarker/Panel of Biomarkers and Levels	Sample Type	Collection Processing (Sample Processing, Check for Haemolysis, Storage Condition)	Study Design: Retrospective/Prospective; Monocenter/Multicenter	Discovery Cohort/Validation Cohort, Histotypes,and Number of Renal Masses	Participant Characteristics,TNM Stage,and Fuhrman Grade	Evaluation Method (RNA Extraction, Retrotrascription, Pre-Amplification, Amplification,and Detection Technology) and Normalization Strategy	Diagnostic/Prognostic Value and Statistical Analysis/Results
[31]	miR-141 ↓, miR-224 ↑, miR-21 ↑, miR-34a ↑	serum	Pre-operative serum samples.	prospective; monocenter	**DC**: 12 RCC (ccRCC); 12 BT; **VC**: NA	**DC**: RCC: Age: 7 pts > 50 y, 5 pts < 50 y; Gender: 8 male; TNM: I: 9 pts, II-III: 3 pts; Fuhrman grade: G1: 3 pts, G2-G3-G4: 9 pts.	Total RNA extraction with mirVana PARIS Kit;RT with PrimeScript One-Step RT-PCR kit (Takara Biomedical Technology); qPCR with SYBR Premix Ex Taq II kit (Takara Biomedical Technology); U6 as endogenous reference gene for normalization.	**diagnostic**: RCC patients vs. benign renal mass patients, *p* < 0.01.
[32]	miR-378 ↑, miR-210 ↑	serum	Pre-operative blood samples, processed within one hour, centrifuged at 1200× *g* for 10 min at 4 °C, and supernatant re-centrifuged at 12,000× *g* for 10 min at 4 °C. Serum stored in liquid nitrogen. Median of storage time: 20 months. For 20 RCC patients, serum samples additionally collected one week and three months after nephrectomy.	prospective; multicenter	**DC**: 195 RCC (157 ccRCC, 12 chRCC, 26 pRCC); 100 CT; **VC**: NA	**DC**: RCC: Age: 64 y; Gender: 133 male; TNM: I-II: 133 pts; III-IV: 62 pts; Fuhrman grade: G1-G2: 115 pts. CT: Age: 52 y; Gender: 65 male.	Total RNA extraction with Qiagen miRNeasy Mini Kit; RT with TaqMan MicroRNA RT Kit (Applied Biosystems); pre-amplification step using TaqMan PreAmp Master Mix; qPCR with TaqMan MicroRNA Assays on a 7500 Real-Time PCR system (Applied Biosystems); data not normalized.	**diagnostic**: AUC = 0.848, sensitivity 80%, specificity 78%.
[33]	miR-378 ↑	serum	Pre-operative blood samples collected in serum S-Monovette Gel tubes with clotting activator. After clotting, serum was separated after centrifugation at 2800× *g* for 10 min and stored in cryotubes at −80 °C.	prospective; multicenter	**DC**: 25 RCC (ccRCC); 25 CT; **VC**: 117 RCC (104 ccRCC, 10 pRCC, 1 chRCC, 2 sRCC); 109 CT; 14 BT.	DC: RCC: Age: 66.4 y; Gender: 12 male; TNM: I-II: 14 pts, III-IV: 11 pts; CT: Age: 61.2 y; Gender: 15 male; VC: RCC: Age: 61.6 y; Gender: 82 male; TNM: I-II: 81 pts; III-IV: 36 pts; CT: Age: 61.9 y; Gender: 75 male; BT: Age: 59.4 y; Gender: 6 male.	Total RNA extraction with mirVana PARIS Kit (Ambion); RT with TaqMan miRNA RT kit (Applied Biosystems) using a self-created Pool of primers from the TaqMan MicroRNA assay for RT; qPCR with TaqMan microRNA assays on a 7900 HT Fast Real-Time PCR System (Applied Biosystems). Cel-miR-39 as exogenous reference gene for normalization.	NA (results not confirmed in the validation cohort).
[34]	miR-122-5p ↑, miR-206 ↑	serum	Pre-operative blood samples were collected in S-Monovette Serum-Gel tubes with clotting activator. After centrifugation, serum was separated and stored in cryotubes at −80 °C.	prospective; monocenter	**DC**: 18 RCC (ccRCC); 8 BT; **VC**: 68 RCC (ccRCC); 47 BT; 28 CT.	**DC**: RCC: Age: 54.6 y; Gender: 12 male; TNM: I-II: 10 pts; III-IV: 8 pts; Fuhrman grade: G1-G2: 6 pts; G3-G4: 11 pts; BT: Age: 64.6 y; Gender: 8 male; **VC**: RCC: Age: 70.4 y; Gender: 48 male; TNM: I-II: 44 pts; III-IV: 24 pts; Fuhrman grade: G1-G2: 47 pts; G3-G4: 21 pts; BT: Age: 65.8 y; Gender: 27 male; CT: Age: 54.6 y; Gender: 15 male.	**DP**: Total RNA extraction with miRNeasy Serum/Plasma kit (Qiagen). Library preparation with NEBNext Small RNA Library Prep Set kit (New England Biolabs); small RNA library pools sequenced on an Illumina NextSeq 500 sequencer; **VP**: Total RNA extraction with mirVana PARIS Kit (Thermo Fisher Scientific); RT with miScript II RT Kit (Qiagen), followed by pre-amplification with the Qiagen miScript PreAMP PCR Kit; qPCR with Qiagen miScript SYBR Green PCR Kit on a 7900 HT Fast Real-Time PCR System (Applied Biosystems);miR-16, miR-191-5p, and miR-320a as endogenous reference genes for normalization.	**prognostic**: correlation with shorter PFS, CSS, and OS; **miR-206 (PFS):** HR = 3.670, 95% CI 1.29–10.51.
[35]	miR-20b-5p ↓, miR-30a-5p ↓, miR-196a-5p ↑	serum	Pre-operative blood samples, processed within 2 h, centrifuged at 3000× *g* for 10 min at 4 °C. Serum stored at −80 °C.	prospective; monocenter	**DC**: 70 RCC (no histologies); 70 CT; **VC**: 40 RCC; 40 CT	**DC**: Age: 50.6 y; Gender: 51 male; Fuhrman grade: G1-G2: 43 pts; G3-G4: 27 pts; CT: Age: 49.6 y; Gender: 45 male; VC: RCC: Age: 49.2 y; Gender: 28 male; Fuhrman grade: G1-G2: 22 pts; G3-G4: 18 pts; CT: Age: 47.9 y; Gender: 25 male.	Total RNA extraction with TRIzol LS reagent (Invitrogen);RT with PrimeScript RT reagent Kit (Takara); qPCR with SYBR Premix Ex Taq II kit (Takara) on LightCycler 480 Real-Time PCR System (Roche Diagnostics); Cel-miR-39 as exogenous reference gene for normalization.	**diagnostic**: AUC = 0.938 (95% CI, 0.889–0.988), sensitivity 92.5%, specificity 80.0%.
[36]	miR-224-5p ↑, miR-34b-3p ↓, miR-182-5p ↓	serum	Blood samples centrifuged at 3000× *g* for 10 min at 4 °C within 2 h. Serum stored at −80 °C.	prospective; monocenter	**DC**: 126 RCC; 130 CT.	**DC**: RCC: Age: 49.4 y; Gender: 66.7% male; TNM: I-II: 20 pts, III-IV: 10 pts; Fuhrman grade: G1-G2: 16 pts, G3-G4: 14 pts; CT: Age: 50 y; Gender: 56.7% male; **VC**: RCC: Age: 50.5 y; Gender: 72.3% male; TNM: I-II: 64 pts, III-IV: 12 pts; Fuhrman grade: G1-G2: 45, G3-G4: 31; CT: Age: 49.8 y; Gender: 63.8% male.	Total RNA extraction with Trizol LS reagent (Invitrogen); RT with PrimeScript RT reagent Kit (TaKaRa) on a general PCR machine (BIO-RAD, USA); qPCR with a SYBR Green qPCR kit (SYBR Pre-mix Ex Taq II, TaKaRa) on LightCycler 480 Real-Time PCR System (Roche Diagnostics). Cel-miR-39 as exogenous reference gene for normalization.	**diagnostic**: AUC = 0.855 (95% CI, 0.797–0.912), sensitivity 80.3%, specificity 66.3%.
[37]	miR-210 ↑	serum	Pre-operative blood samples. Serum was separated after centrifugation at 3000 rpm for 10 min and stored at −80 °C.	prospective; monocenter	**DC**: 34 RCC (ccRCC); 23 CT; **VC**: NA	**DC**: RCC: Age: 66.5 y; Gender: 26 male; TNM: I-II: 29; III-IV: 5; Fuhrman grade: G1-G2: 33 pts; G3: 1 pts. CT: Age: 53.5 y; Gender: 11 male.	Total RNA extraction with microRNA extractor SP kit (Wako, Japan); RT with TaqMan miRNA RT kit (Applied Biosystems); qPCR with TaqMan microRNA assays on a 7900 HT Fast Real-Time PCR System (Applied Biosystems); miR-16 as endogenous reference gene for normalization.	**diagnostic**: AUC = 0.77 (95% CI, 0.65–0.89), sensitivity 65%, specificity 83%.
[38]	miR-21-5p ↑,miR-210-3p ↓	serum	preoperative serum samples; tested for haemolysis.	prospective; multicenter	**DC**: 67 RCC (34 pRCC I, 33 pRCC II); 33 CT; **VC**: NA	**DC**: pRCC1: Age: 63.6 y; Gender: 30 male; TNM: I-II: 31 pts; III-IV: 3 pts; Fuhrman grade: G1-G2: 32 pts; G3-G4: 2 pts; pRCC2: Age: 67.4 y; Gender: 28 male; TNM: I-II: 28 pts; III-IV: 5 pts; Fuhrman grade: G1-G2: 24 pts; G3-G4: 9 pts.	RNA extraction and RT-qPCR executed by Exiqon RNA services;the geometric mean of miR-23a-3p, miR-191-5p and miR-103a-3p was used for normalization.	**prognostic (localized vs. advanced pRCC)**: AUC = 0.718.
[39]	miR-508-3p ↓, miR-885-5p ↑	serum	Blood samples centrifuged at 2860× *g* for 10 min. Serum stored at −80 °C.	prospective; monocenter	**DC**: 10 RCC (ccRCC); 10 CT; **VC**: 85 RCC (ccRCC); 35 CT.	**DC**: RCC: Age: 44 > 60 y; Gender: 45 male; TNM: I-II: 63 pts; III-IV: 22 pts; CT: Age: 16 > 60 y; **VC**: RCC: Age: 41 < 60 y; CT: 17 < 60 y.	Total RNA extraction with Trizol LS reagent (Invitrogen); RT-qPCR with Hairpin-it microRNA RT-PCR Quantitation kit (GenePharma). Cel-miR-39 as exogenous reference gene for normalization.	**diagnostic**: AUC = 0.90 (95% CI: 0.84-0.96).
[40]	miR-210 ↑	serum	peripheral venous blood was extracted from the renal carcinoma patients and healthy subjects, stood, and centrifuged. The serum was separated.	prospective; monocenter	**DC**: 32 RCC (ccRCC); 32 CT; **VC**: NA.	**DC**: RCC: Age: 62 y; Gender: 25 male; CT: 61 y; Gender: 22 male.	Total RNA extraction with Trizol reagent (Invitrogen); RT-qPCR with the Bulge-LoopTM miRNA RT-qPCR Primer Set; RT with Promega Reverse Transcription kit (Promega); qPCR on a 7900 HT Fast Real-Time PCR System (Applied Biosystems). U6 as endogenous reference gene for normalization.	**diagnostic**: *p* < 0.001.
[41]	miR-378 ↑, miR-451 ↓	serum	Pre-operative RCC serum samples.	prospective; monocenter	**DC**: 15 RCC (ccRCC); 12 CT; **VC**: 90 RCC (73 ccRCC, 8 pRCC, 9 chRCC); 35 CT.	**DC**: RCC: Age: 62 y; Gender: 10 male; TNM: I-II: 6 pts; III-IV: 9 pts; Fuhrman grade: G1-G2: 10 pts, G3-G4: 5 pts; CT: Age: 61 y; Gender: 10 male; **VC**: RCC: Age: 66 y; Gender: 56 male; TNM: I-II: 64 pts; III-IV: 26 pts; Fuhrman grade: G1-G2: 59 pts; G3-G4: 30 pts; CT: Age: 63 y; Gender: 26 male.	Total RNA extracted with miRNeasy Mini Kit (Qiagen). **DP**: RT with TaqMan miRNA RT kit and Megaplex RT primers, followed by a pre-amplification step with TaqMan PreAmp MasterMix (Applied Biosystems); qPCR with TaqMan Low Density Arrays on a 7900 HT Fast Real-Time PCR System (Applied Biosystems). **VP**: RT with TaqMan miRNA RT kit (Applied Biosystems); qPCR with TaqMan microRNA assays on a 7500 Real-Time PCR system (Applied Biosystems). MiR-16 as endogenous reference gene for normalization.	**diagnostic**: AUC = 0.86, sensitivity 81%, specificity 83%.
[42]	miR-21 ↑, miR-106a ↑	serum	Pre-operative blood samples, collected in serum S-Monovette Gel tubes with clotting activator, and processed within five hours. After clotting, serum was separated after centrifugation at 300× *g* for 10 min and stored in cryotubes at −80 °C.	prospective; monocenter	**DC**: 30 RCC (ccRCC); 30 CT.	**DC**: RCC: Age: 52 y; Gender: 23 male; Fuhrman grade: I-II: 18 pts, III: 12 pts. CT: Age: 47 y; Gender: 21 male.	Total RNA extraction with mirVana PARIS Kit; RT with RT Kit (Thermo Scientific) with miRNA-specific RT primers synthesized by Sangon Biotech; qPCR with Maxima SYBR Green qPCR Kit (Thermo Scientific) on a7500 Real-Time PCR system (Applied Biosystems); U6 as endogenous reference gene for normalization.	**diagnostic**: AUC (miR-21) = 0.865 (95% CI: 0.766–0.965), sensitivity 77.3%, specificity 96.4%; AUC (miR-106a) = 0.819 (95% CI: 0.710–0.929), sensitivity 86.7%, specificity 70.0%.
[44]	miR-200a ↓	serum	Pre-operative blood samples collected after 12 h of overnight fasting, immediately centrifuged at 1500× *g* for 10 min at R.T., and supernatant centrifuged at 12,000× *g* for 5 min at 4 °C. Serum samples stored at −80 °C.	prospective; monocenter	**DC**: 26 RCC (ccRCC); 26 CT; **VC**: 73 RCC (ccRCC); 73 CT.	**DC**: RCC: Age: 53.7 y; Gender: 20 male; TNM: I-II: 22 pts; III-IV: 3 pts; CT: Age: 51.7 y; Gender: 14 male; **VC**: RCC: Age: 52.7 y; Gender: 21 male; TNM: I-II: 63 pts; III-IV: 7 pts; CT: Age: 54.9 y; Gender: 30 male.	Total RNA extraction with a 1-step phenol/chloroform purification protocol; RT with TaqMan miRNA RT kit (Applied Biosystems);qPCR with TaqMan microRNA assays on a 7500 Real-Time PCR system (Applied Biosystems); combination of let-7d, let-7g, and let-7i (let-7d/g/i) as endogenous reference genes for normalization.	**diagnostic**: RCC vs. CRT: AUC = 0.702 (95% CI, 0.618–0.785); stage I RCC vs. CRT: AUC = 0.740 (95% CI, 0.667–0.814); stage II RCC vs. CRT: AUC = 0.700 (95% CI, 0.544–0.847); stage I-II vs. CRT: AUC = 0.733 (95% CI, 0.662–0.804).
[43]	miR-193a-3p ↑, miR-362 ↑, miR-572 ↑,miR-28-5p ↓, miR-378 ↓	serum	Pre-operative blood samples collected after 12 h of overnight fasting, immediately centrifuged at 3000× *g* for 5 min at R.T., and supernatant centrifuged at 10,000× *g* for 5 min at 4 °C. Serum samples stored at −80 °C. The storage time rangedfrom 5 days to 334 days (mean ± SD: 213 ± 122 days).	prospective; monocenter	**DC**: 25 RCC (ccRCC); 25 CT; **VC**: 107 RCC (ccRCC); 107 CT.	**DC/VC**: RCC: Age: 53.5 y; Gender: 79 male; TNM: I-II: 92 pts, III-IV: 10 pts; CT: Age: 53.7 y; Gender: 69 male.	**DP**: Total RNA extraction with TRIzol reagent (Invitrogen); RT with TaqMan miRNA RT kit and Megaplex RT primers, followed by a pre-amplification step with TaqMan PreAmp MasterMix (Applied Biosystems); qPCR with TaqMan Low Density Arrays on a 7900 HT Fast Real-Time PCR System (Applied Biosystems); data normalized to an internal control; **VP**: Total RNA extraction with a 1-step phenol/chloroform purification protocol; RT with TaqMan miRNA RT kit (Applied Biosystems);qPCR with TaqMan microRNA assays on a 7500 Real-Time PCR system (Applied Biosystems); combination of let-7d, let-7g, and let-7i (let-7d/g/i) as endogenous reference genes for normalization.	**diagnostic**: RCC vs. CRT: AUC = 0.796 (95% CI, 0.724–0.867); early stage ccRCC vs. CRT: AUC = 0.807 (95% CI, 0.731–0.871), sensitivity 80%, specificity 71%.
[45]	miR-429 ↑	serum	NA	prospective; monocenter	**DC**: 27 RCC; 28 CT; **VC**: NA	**DC**: RCC: Age: 42.3 y; Gender: 15 male; CT: 44.1 y; Gender: 14 male.	NA	both **diagnostic and prognostic**.
[46]	miR-1233 ↑	serum	Pre-operative blood samples, collected in serum S-Monovette Gel tubes with clotting activator and processed between 1 and 3 h. After clotting, serum was separated after centrifugation at 2800× *g* for 10 min and stored in cryotubes at −80 °C.	prospective; monocenter	DCI: 6 RCC (ccRCC); 6 CT; DCII: 33 RCC (ccRCC); 30 CT; VC: 84 RCC (69 ccRCC, 10 pRCC, 3 chRCC, 2 sRCC); 93 CT; 13 BT.	**DCI**: RCC: Age: 66 y; Gender: 3 male; TNM: I-II: 3 pts; III-IV: 3 pts; Fuhrman grade: G1-G2: 6 pts; G3-G4: 0 pts; CT: Age: 47 y; Gender: 3 male; **DCII**: RCC: Age: 62.7 y; Gender: 27 male; TNM: I-II: 26 pts; III-IV: 7 pts; Fuhrman grade: G1-G2: 32 pts; G3-G4: 1 pts; CT: Age: 60.2 y; Gender: 21 male; VC: RCC: Age: 60.9 y; Gender: 56 male; TNM: I-II: 59 pts; III-IV: 25 pts; Fuhrman grade: G1-G2: 73 pts; G3-G4: 11 pts; CT: Age: 63.7 y; Gender: 68 male; BT: Age: 59.5 y; Gender: 6 male.	Total RNA extraction with mirVana PARIS Kit; **DP**: RT with TaqMan miRNA RT kit and Megaplex RT primers, followed by a pre-amplification step with TaqMan PreAmp MasterMix (Applied Biosystems); qPCR with TaqMan Low Density Arrays on a 7900 HT Fast Real-Time PCR System (Applied Biosystems). **VP**: RT with TaqMan miRNA RT kit (Applied Biosystems), using a self-created Pool of primers from the TaqMan MicroRNA assay for RT; qPCR with TaqMan microRNA assays on a 7500 Real-Time PCR system (Applied Biosystems). Cel-miR-39 as exogenous reference gene for normalization.	**diagnostic**: AUC of 0.588 (95% CI 0.505–0.671), sensitivity 77.4%, specificity 37.6%; no difference between RCC patients and patients with benign renal mass.
[47]	miR-141 ↓,miR-1233 ↑	serum	Pre-operative blood samples processed immediately, and serum stored at −80 °C.	prospective; monocenter	**DC**: 30 RCC (ccRCC); 15 CT; **VC**: NA	**DC**: RCC: Age: 54 y; Gender: 76.6% male. TNM: I-II: 23 pts; III-IV: 7 pts.	Total RNA extraction with miRNA Serum/Plasma kit (Qiagen); RT with miScript II RT Kit (Qiagen); qPCR with miScript SYBR Green PCR Kit (Qiagen) on a BIORAD CFX 96 Machine (Hercules, California). Cel-miR-39 as exogenous reference gene for normalization.	**diagnostic**: 100% sensitivity, 73% specificity.
[48]	miR-183 ↑	serum	Pre-operative blood samples, 1 h coagulation at R.T., centrifugation at 820× *g* for 10 min at 4 °C, and supernatant centrifugation at 16,000× *g* for 10 min at 4 °C.	prospective; monocenter	**DC**: 82 RCC; 19 CT; **VC**: NA	Not specified	Total RNA extraction with TRIzol reagent (Invitrogen);RT with TaKaRa microRNA transcription kit (Takara); qPCR with SYBR Premix Ex Taq II kit (Takara) on an ABI-7300 Real-Time PCR System (Applied Biosystems); U6 as endogenous reference gene for normalization.	**diagnostic**: *p* < 0.01; level of miR-183 **positively correlated with the grading of RCC** (R = 0.611, *p* < 0.01); **predictive biomarker** of the response of RCC cells to the cytotoxicity induced by NK cells (response rates inversely associated with the levels of serum miR-183 (R = −0.608, *p* < 0.01).
[49]	miR-210↑	serum	Pre-operative blood samples, processed within 5 h, centrifuged at 1200× *g* for 10 min at 4 °C, and supernatant centrifuged at 10,000× *g* for 10 min at 4 °C. Serum stored at −80 °C. For 10 RCC patients, serum samples additionally collected 7 days after surgery.	prospective; monocenter	**DC**: 68 RCC (ccRCC); 42 CT; **VC**: NA	**DC**: RCC: Age: 64 y; Gender: 46 male; TNM: I-II: 31 ptd; III: 37 pts; Fuhrman grade: G1-G2: 41 pts, G3-G4: 27 pts. CT: Age: 59 y, Gender: 22 male.	Total RNA extracted with MicroMini Kit (Qiagen); RT with miScript Reverse Transcription Kit (Qiagen); qPCR with miScript SYBR Green PCR kit (Qiagen) on a 7500 Real-Time PCR system (Applied Biosystems); 5s rRNA as endogenous reference gene for normalization.	**diagnostic**: AUC = 0.874, sensitivity 81.0%, specificity of 79.4%.
[50]	miR-625-3p ↓	serum	Pre-operative blood samples centrifuged at 3000 rpm for 10 min. Serum stored at −80 °C.	prospective; monocenter	**DC**: 50 RCC (ccRCC); 74 CT; **VC**: NA	**DC**: RCC: Age: 49.5 y; Gender: 35 male; TNM: I-II: 49 pts, III-IV: 1 pts; CT: 54.3 y; Gender: 34 male.	Total RNA extraction with miRNeasy Serum/Plasma Kit (Qiagen); RT with miScript II RT Kit (Qiagen); qPCR with miScript SYBR^®^ Green PCR kit (Qiagen) on a Roche Lightcycler 480 Real-Time PCR system (Roche Diagnostics). Cel-miR-54 as exogenous reference gene for normalization.	**diagnostic**: AUC = 0.792 (95% CI, 0.714–0.870), sensitivity 70.3%, specificity 80.0%.
[51]	miR-150 ↓	plasma	Pre-operative blood collected in EDTA tubes and processed within 2 h. Plasma separated after centrifugation at 2000× *g* for 10 min at R.T. Plasma stored at −80 °C.	prospective; multicenter	**DC**: 94 RCC (ccRCC); 100 CT; **VC**: NA	**DC**: RCC: Age: 57.8 y; Gender: 51 male; TNM: I-II: 51 pts, III-IV: 43 pts; CT: Age: 60.1 y; Gender: 71 male.	Total RNA extraction with NucleoSpin miRNA Plasma kit (Macherey-Nagel); RT-qPCR with TaqMan Low Density Arrays on a 7900 HT Fast Real-Time PCR System (Applied Biosystems). Quantile normalization.	**prognostic (DSS)**: HR = 1.3, 95% CI 1.0–1.8, *p* = 0.03.
[52]	miR-210 ↑, miR-224 ↑	plasma	Pre-operative and post-operative day-7 blood samples collected in EDTA tubes and processed within 1 h of collection by centrifugation at 820× *g* at 4 °C for 10 min, and supernatant centrifuged at 16,000× *g* at 4 °C for 10 min. Plasma stored at −80 °C. Samples exhibiting evidence of hemolysis excluded.	prospective; monocenter	**DC**: 66 RCC (ccRCC); 67 CT; **VC**: NA	**DC**: RCC: Age: 56 y; Gender: 39 male; TNM: I-II: 64 pts, III-IV: 2 pts; Fuhrman Grade: G1-G2: 44 pts, G3-G4: 22 pts. CT: matched.	Total RNA extraction with TRI Reagent BD (Molecular Research Center); RT with RevertAid First Strand cDNA Synthesis Kit (Thermo) and a RT primer from Guangzhou RiboBio Co., Ltd.; RT-qPCR with Platinum SYBR Green qPCR Supermix UDG (Invitrogen), and primers synthesized at Guangzhou RiboBio Co., Ltd. on a LightCycler 480 II (Roche Diagnostics). Cel-miR-39 as exogenous reference gene for normalization.	**diagnostic**: AUC (miR-210) = 0.6775, sensitivity 89.55%, specificity 48.48%; AUC (miR-224) = 0.6056, sensitivity 88.06%, specificity 40.91%; AUC (miR210 × 224) = 0.6592, sensitivity 92.54%, specificity 45.45%.
[53]	miR-210 ↑, miR-221 ↑, miR-1233 ↑	plasma	Peripheral venous blood samples collected in EDTA tubes and centrifuged for 5 min at 3000 rpm at R.T.	prospective; monocenter	**DC**: 54 RCC (39 ccRCC, 15 others); 50 CT; **VC**: NA	**DC**: RCC: Age: 60.3 y; Gender: 40 male: TNM: I-II: 19 pts, III-IV: 31 pts; Furhman Grade: G1-G2: 16 pts, G3-G4: 35 pts. CT: Age: 43 y, Gender: 16 male.	Total RNA extraction with an acid phenol-chloroform (5:1) solution (Ambion^®^) and miRNA purification with GRS microRNA kit (Grisp); RT with TaqMan MiRNA RT Kit (Applied Biosystems); qPCR with TaqMan microRNA assays on a StepOne Real-Time PCR System (Applied Biosystems). RNU48 as endogenous reference gene for normalization.	**prognostic (CSS)**: HR = 3.02, 95% CI 1.19–7.64, *p* = 0.014.
[54]	miR-144-3p ↑	plasma	Pre-operative blood samples and post-operative 7-day blood samples collected in EDTA tubes, processed within 2 h, centrifuged at 800× *g* for 10 min at 4 °C, and supernatant centrifuged again at 12,000× *g* for 15 min at 4 °C. Plasma stored at −80 °C.	prospective; monocenter	**DC**: 106 RCC (ccRCC); 28 BT; 123 CT.	**DC**: RCC: Age: 58 < 60 y; 48 > 60 y; Gender: 74 male; TNM: I-II: 66 pts, III-IV: 38 pts; Fuhrman grade: G1-G2: 77 pts, G3-G4: 27 pts.	**DP**: The miRNA expression profiling with the Agilent Human miRNA microarray 18.0 (Agilent Technologies). **VP**: Total RNA extraction with TRI Reagent BD (Molecular Research Center); RT with RevertAid First-Strand cDNA Synthesis Kit (Thermo) with RT primers from RiboBio; qPCR with SYBR Green mix (Thermo) and primers obtained from RiboBio. MiR-320c and cel-miR-39 as an endogenous and an exogenous reference gene for normalization, respectively.	**diagnostic**: ccRCC vs. CRT: AUC of 0.91 (95% CI, 0.88–0.95), sensitivity 87.1%, specificity 83.02%; ccRCC vs. angiomiolipoma patients: AUC 0.82 (95% CI, 0.74–0.91), sensitivity 75%, specificity 71.7%.
[55]	miR-7 ↑, miR-221 ↑, miR-222 ↑	plasma	Pre-operative peripheral venous blood samples.	prospective; monocenter	**DC**: 22 RCC; 27 CT; **VC**: NA.	**NA**	Total RNA extraction with GRS microRNA Kit (GRISP); RT TaqMan MiRNA RT Kit (Applied Biosystems); qPCR with TaqMan microRNA assays on a StepOne Real-Time PCR System (Applied Biosystems). RNU48 as endogenous reference gene for normalization.	**prognostic**; miR-7,miR-221 and miR-222 plasma levels could be useful phenotype biomarkers of EGFR/MAPK activation.
[56]	miR-221 ↑	plasma	Plasma separation from peripheral venous blood samples by centrifugation.	prospective; monocenter	**DC**: 43 RCC (37 ccRCC, pRCC, 12 chRCC); 34 CT; **VC**: NA.	**DC**: RCC: Age: 60.1 y; Gender: 32 male; TNM: I-II: 24 pts, III-IV: 19 pts; Furhman Grade: G1-G2: 15 pts, G3-G4: 9 pts; CT: Age: 50.9 y; Gender: 19 male.	Total RNA extraction with mirVana PARIS Kit (Ambion); RT TaqMan MiRNA RT Kit (Applied Biosystems); qPCR with TaqMan microRNA assays on a StepOne Real-Time PCR System (Applied Biosystems). RNU44 as endogenous reference gene for normalization.	**prognostic (OS)**: *p* = 0.024; **prognostic (CSS)**: HR = 10.7, 95% CI 1.33–85.65, *p* = 0.026.
[58]	miR-483-5p ↓	plasma	pre-operative and post-operative 7-day plasma samples.	prospective; monocenter	**DC**: 12 RCC (ccRCC).	Not specified	NA	**diagnostic**
[57]	miR-508-3p ↓	plasma	Plasma separated from whole blood and stored at −80 °C after the addition of TRIzol reagent (Invitrogen).	prospective; multicenter	**DC**: 50 RCC (36 ccRCC, 6 pRCC, 8 chRCC); **VC**: NA	**DC**: RCC: Age: 23< 52 y; 27 > 52 y; Gender: 28 male; TNM: I-II: 45 pts, III-IV: 5 pts; NO CT.	RT with miScript Reverse Transcription (Qiagen); qPCR with miScript SYBR Green PCR Kit (Qiagen) on an ABI PRISM 7000 Real-Time PCR System (Applied Biosystems).	**diagnostic**: *p* < 0.01
[59]	miR-187 ↓	plasma	Pre-operative peripheral venous blood samples; plasma stored at −80 °C.	prospective; monocenter	**DC**: 108 RCC (ccRCC); 50 CT; **VC**: NA	Not clear: description of only 54 pts.	Total RNA extraction with TRIzol (Invitrogen); RT with M-MLV RT (Promega); qPCR with SYBR green I mix (Takara) on an ABI PRISM 7000 Real-Time PCR System (Applied Biosystems).	**diagnostic**
[62]	miR-210 ↑,miR-1233 ↑	serum EVs	Blood sample collected 1 d before tumor resection. Serum samples obtained via centrifugation at 1200× *g* for 10 min at 4 °C. The supernatant collected and further centrifuged at 12,000× *g* for 10 min at 4 °C to completely remove cellular components. The cell-free serum samples were then stored at −80 °C until exosome isolation.	monocenter; retrospective	**DC**: 82 RCC (ccRCC); 80 CT.	**DC**: RCC: Age: 41 > 58 y; 41 < 57 y; Gender: 42 male; TNM: I-II: 52 pts, III: 30 pts; Fuhrman Grade: G1-G2: 62 pts, G3-G4: 20 pts.	Total RNA extracted with MicroMini kit (Qiagen); RT with miScript RT kit (Qiagen); qPCR with miScript SYBR green PCR kit (Qiagen) on a 7500 qPCR system (Applied Biosystems).	**diagnostic**. AUC (miR-210) = 0.69, sensitivity 70%, specificity 62.2%; AUC (miR-1233) = 0.82, sensitivity 81%, specificity 76%.
[63]	miR-210 ↑	serum EVs	The samples were centrifuged at 1000× *g* for 10 min at 4 °C. The supernatants were further centrifuged at 10,000× *g* for 10 min at 4 °C to completely eliminate cellular components. The serum was stored at −80 °C before analysis.	monocenter; retrospective	**DC**: 45 RCC (ccRCC, 5 metastatic); 30 CT.	**DC**: RCC: Age: 10 < 50 y, 35 > 50 y; Gender: 29 male; TNM: I-II: 28 pts, III-IV: 17 pts; Fuhrman Grade: G1-G2: 26 pts, G3-G4: 19 pts; 5 metastatic. CT: matched.	Total RNA extracted with TRIzol reagent (Invitrogen); RT with the Reverse Transcriptase M-MLV (Invitrogen); qPCR with SYBR Green PCR master mix (Invitrogen) on a qPCR LightCycler480 System (Roche).	**diagnostic/prognostic**. AUC = 0.8779, 67.5% sensitivity and 70.0% specificity.
[64]	miR-149-3p ↑, miR-424-3p ↑, miR-92a-1-5p ↓	plasma EVs	Blood collected from the elbow vein with an EDTA-K2 tube. Each sample centrifuged at 3000 rpm for 10 min at 4 °C, and the isolated plasma samples were stored at −80 °C until RNA isolation.	monocenter; prospective	**DC**: 5 RCC (18 ccRCC, 4 pRCC); 5 CT: **VC**: 22 RCC, 16 CT.	VC: RCC: Age: 56 y; Gender: 13 male;Fuhrman Grade: G1-G2: 13 pts; G3-G4: 9 pts.	Total RNA extracted with exoEasy kit columns (exoRNeasy Serum kit protocol) (QIAGEN). NGS libraries prepared with QIAseq miRNA Library Kit (QIAGEN). Sequencing performed on an Illumina NovaSeq 6000 System, and data analyzed with the QIAseq miRNA quantification platform using unique molecular index counts.	**diagnostic**. AUC (miR-149-3p) = 0.7188, specificity 75%, sensitivity 72.7%; AUC (miR-424-3p) = 0.7727, specificity 75%, sensitivity 81.8%; were upregulated, while those of AUC (miR-92a-1-5p) = 0.8324, specificity 87.5%, sensitivity 77.3%.
[65]	miR-224 ↑	serum EVs	Blood sampling was performed before surgery.	monocenter; prospective-retrospective	**DC**: 108 RCC (ccRCC).	**DC**: RCC: Age: 64.5 y; Gender: 67 male; TNM: I-II: 81 pts, III-IV: 27 pts; Fuhrman Grade: G1-G2: 76 pts, G3-G4: 32 pts.	MiRNAs isolated from exosomes with Total Exosome RNA and Protein Isolation kit (Life Technologies) and qRT-PCR performed with TaqMan universal PCR master mix on StepOnePlus Real-Time PCR System (Applied Biosystems). RNU48 and miR-16 as endogenous reference gene for normalitazion.	**prognostic**. High exosomal miR-224 expression was a significant independent risk factor related to PFS, CSS, and OS in multivariate analysis (HR = 11.0; *p* < 0.0001, HR = 1.6; *p* = 0.0140, HR = 9.1; *p* = 0.0043). RCC patients vs. healthy controls, *p* < 0.0001.
[66]	miR-let-7i-5p ↑, miR-26a-1-3p ↑, miR-615-3p ↑	plasma EVs	Plasma was collected, uniformly processed, and stored at −80 °C before use.	monocenter; prospective	**DC**: 44 RCC (40 ccRCC, 2 pRCC, 2 unspecified); **VC**: 65 RCC (52 ccRCC, 2 chRCC, 6 pRCC, 5 unspecified).	**DC**: Age: 70.2 y; Gender: 35 male; TNM: I-II: 13 pts, III-IV: 20 pts; Fuhrman Grade: G1-G2: 16 pts, G3-G4: 23 pts;**VC**: RCC: Age: 64.6 y; Gender: 48 male. TNM: I-II: 17 pts, III-IV: 39 pts; Furhman Grade: G1-G2: 21 pts, G3-G4: 33 pts.	Total RNA extracted with miRNeasy Micro Kit (QIAGEN). **DP**: RNA libraries prepared with NEBNext Multiplex Small RNA Library Prep Set (NEB); 50 bp single read sequencing using Illumina HiSeq2000 DNA sequence analyzer; **VP**: RT with TaqMan Advanced miRNA cDNA Synthesis Kit; qPCR on CFX384 Real-Time PCR Detection System (BIO-RAD);miR-127-3p as endogenous reference gene for normalitazion.	**prognostic**. OS association: miR-let-7i-5p (*p* = 0.018, HR = 0.49, 95% CI = 0.21–0.84), miR-26a-1-3p (*p* = 0.025, HR = 0.43, 95% CI = 0.10–0.84), and miR-615-3p (*p* = 0.0007, HR = 0.36, 95% CI = 0.11–0.54).
[67]	miR-301a-3p ↑, miR-1293 ↓	plasma EVs	All blood collections were performed during the morning period and stored at 4 °C immediately until sample processing.	monocenter; prospective	**DC**: 69 RCC (32 ccRCC, 37 metastatic).	**DC**: localized RCC: Age: 61.9 y; Gender: 24 male; TNM: I-II: 18 pts, III-IV: 13 pts; Fuhrman grade ISUP: G1-G2: 20 pts, G3-G4: 12 pts; Advanced RCC: Age: 62.4 y; Gender: 26 male: TNM: I-II: 16 pts, III-IV: 19 pts; Fuhrman grade ISUP: G1-G2: 15 pts, G3-G4: 17 pts.	MiRNA isolated from EVs with the Plasma/Serum RNA Purification Mini Kit (NORGEN). RT with TaqMan Advanced miRNA cDNA Synthesis Kit (Applied Biosystems). qPCR with TaqMan Advanced miRNA Assays probes on StepOnePlus Real-Time PCR System (Applied Biosystems); hsa-let7a-5p and hsa-miR-16-5p as endogenous reference genes for normalitazion.	**prognostic**. Localized disease vs. metastatic higher miR-301a-3p (*p* = 0.026) and lower levels of miR-1293 (*p* = 0.004).
[68]	miR-126-3p↓	urine EVs	Urine collected from each individual, centrifuged at 2000× *g* for 10 min at 4 °C, and stored at −80 °C until use.	prospective; monocenter	**DC**: 28–30 RCC (ccRCC); 18 CT. **VC**: 81 RCC (ccRCC); 24 BT; 33 CT.	**DC**: RCC: Age: 59 y; Gender: 18 male;TNM; I-II: 26 pts; III-IV: 3 pts, Fuhrman Grade; G1-G2: 19 ptsG3-G4: 9 pts. **VC**: non specified.	MiRNA extracted with the Norgen kit. RT with Custom Made Megaplex RT primer Pool and TaqMan MicroRNA RT Kit (Life Technologies); pre-amplification step using TaqMan PreAmp Master Mix; qPCR on ViiA7 Real Time PCR System (Life Technologies); miR-16-5p and miR-106a-5p as endogenous reference genes for normalitazion.	**diagnostic**. MiR-126-3p–miR-449a ccRCC vs. controls: AUC = 0.84; 95% CI, *p* < 0.001; miR-126-3p–miR-34b-5p: AUC = 0.79; 95% CI, *p* < 0.001. MiR-126-3p-miR-34b-5p small renal masses (pT1a, 4 cm) vs. healthy controls: AUC: 0.79; 95% CI, *p* < 0.001. MiR-126-3p-miR-486-5p benign lesions vs. ccRCC: AUC: 0.85; 95% CI, *p* < 0.01.
[69]	miR-30c-5p ↓	urine EVs	Morning urine was collected and centrifuged (2000× *g* for 5 min) at 4 °C and then filtrated at 0.22 μm before being stored at −80 °C.	prospective; multicenter	**DC**: 70 RCC (ccRCC); 30 CT.	**DC**: RCC: Age: 55 y; Gender: 35 male; TNM: I-II: 70 pts; III-IV: 0 pts. CT: Age: 51 y; Gender: 15 male.	**DP**: total RNA extracted with a TRIzol Plus RNA Purification Kit (Life Technologies). Small RNA enriched from total RNA by increasing the ethanol content of the sample, followed by isolation over a glass-fibre filter and elution; Sequencing performed on a HiSeq 2000 (Illumina); **VP**: RT-qPCR with TaqMan MicroRNA Assay (Life Technologies).	**diagnostic**. AUC = 0.8192 (95% CI: 0.7388–0.8996, *p* < 0.01) with 68.57% sensitivity and 100% specificity.
[70]	miR-224-5p↑	urine EVs	Urine samples collected prior to surgery, delivered to the laboratory on ice, and processed to isolate EVs promptly or stored at −80 °C for further use.	prospective; monocenter	**DC**: 6 RCC; 6 CT.	**DC**: RCC: Age: 61,43 y; Gender: 5 male; TNM: I-II: 6 pts; III-IV: 0 pts; CT: 50,6 y: Gender: 4 male.	Total RNA extracted with miRNeasyTM Micro kit; RT with random primers and a PrimeScriptTM RT Master Mix kit (Takara). Specific stem-loop primers used to detect miRNAs by an miRNA 1st Strand cDNA Synthesis kit (Vazyme). qPCR withTB Green Premix Ex TaqTM (Takara) on a Real-Time PCR System (Applied Biosystems). GAPDH or RNU6-1 (U6) as endogenous reference genes for normalitazion.	**diagnostic**
[71]	Circ_400068 ↑	plasma EVs	Circulating blood samples centrifugated at 2000× *g* at 4 °C for 10 min, and the plasma was obtained and processed to isolate EVs.	prospective; monocenter	**DC**: 28 RCC.	**DC**: RCC: Age: 18 > 60 y, 10 < 60 y; Gender: 16 male; Fuhrman Grade: G1-G2: 17 pts, G3-G4: 11 pts.	Total RNA extracted with TRIzol^®^ reagent (Invitrogen). RT with PrimeScript™ RT kit (Takara Biotechnology). qPCR with SYBR Green PCR Master mix (Takara Biotechnology). GAPDH as endogenous reference gene for normalitazion.	**diagnostic**
[72]	miR-122 ↑, miR-1271 ↑, miR-15b	urine EVs	Urine samples collected the day before the surgery. Urine stabilized within 4 h using a urine preservative (Norgen Biotek) and stored at 4 °C. Urine samples were not centrifuged before RNA extraction.	prospective; monocenter	**DC**: 17 RCC (ccRCC); 14 CT.	**DC**: RCC: Age: 63.71 y; Gender: 13 male; TNM: II-II: 10 pts, III-IV: 7 pts; Fuhrman grade: G1-G2: 12 pts, G3-G4: 5 pts; CT: Age: 57.14 y; Gender: 10 male.	Total RNA extracted with miRNeasy micro kit (Qiagen); RT with the miScriptII RT kit (Qiagen) containing miRTC (Qiagen); preamplification with miScriptPreAMP PCR kit (Qiagen); RT-qPCR with miScriptSYBR Green PCR kit (Qiagen) on ABI7300 cycler (Thermo-Fisher).	**diagnostic**. 7p-urinary score: AUC = 0.96, with 100% sensitivity, and 86% specificity.
[73]	miR-328-3p ↓	urine EVs	NA	prospective; monocenter	**DC**: 6 RCC (ccRCC); 3 BT; **VC**: 44 RCC (ccRCC); 27 BT.	**DC/VC**: RCC: Age: 20 < 81 y; 24 > 81 y; Gender: 29 male; Fuhrman grade: G1-G2: 35 pts; G3-G4: 1 pts. CT: matched.	Total RNA extracted with miRNeasy serum/plasma kit (Qiagen). RT with the TaqMan miRNA RT Kit (Applied Biosystems); pre-amplification step using TaqMan PreAmp Master Mix; qPCR on the Viia7 Real-Time PCR System (Applied Biosystems).	**prognostic**. AUC: 0.68, 95% CI: 0.52 to 0.84, *p* = 0.038
[74]	let-7a ↑, let-7b ↑, let-7c ↑, let-7d ↑, let-7e ↑, let-7g ↑	urine EVs	The first morning urine samples collected with EDTA and kept at 4 °C till further processing. Urine sample centrifuged at 4 °C at 2000× *g* for 15 min, and the cell-free supernatant was then collected and stored at −80 °C until analysis.	prospective; monocenter	**DC**: 69 RCC (ccRCC); CT: 36.	**DC**: RCC: Age: 66 y; Gender: 50 male; TNM: I-II: 58 pts; III-IV: 11 pts; Fuhrman grade: G1-G2: 51 pts; G3-G4: 18 pts. CT: Age: 65 y. Gender: 24 male.	Total RNA extracted with manual column-based method, urine miRNA Purification Kit (Norgen Biotek). RT with miRNA-specific stem-loop RT primer and the TaqMan^®^ MicroRNA RT Kit (Thermo Fisher Scientific). qPCR performed with TaqMan MicroRNA assays on Roche LightCycler 480 PCR system (Roche).	**diagnostic**. AUC = 0.8307 with 71% sensitivity and 81% specificity.
[75]	miR-210 ↑	urine EVs	Cell-free urine samples obtained by the initial centrifugation of the whole urine samples at 1200× *g* for 10 min at 4 °C, followed by centrifugation at 12,000× *g* for 10 min at 4 °C. Cell-free urine samples stored at−80 °C until use. Urine samples were processed within 6 h of the urine draw.	prospective; monocenter	**DC**: 75 RCC (ccRCC); 45 CT. 15 RCC after surgery	**DC**: RCC: Age: 64 y; Gender: 54 male; TNM: I-II: 52 pts, III: 23 pts; Fuhrman grade: G1-G2: 36 pts, G3-G4: 39 pts. CT: Age: 60 y; Gender: 25 male.	Total RNA extracted with MicroMini Kit (Qiagen). RT with miScript RT Kit (Qiagen). qPCR with miScript SYBR Green PCR kit (Qiagen) on a on a 7500 qPCR system (Applied Biosystems).	**diagnostic**. AUC = 0.76 with 57.8% sensitivity and 80.0% specificity.
[76]	miR-15a ↓	urine EVs	Urine collected and stored at −20 °C until further examination.	prospective; monocenter	**DC**: 67 RCC (22 ccRCC; 16 pRCC; 14 chRCC); 15 BT; 15 CT.	**DC**: RCC: Age: 60.7 y; Gender: 32 male; TNM: I-II: 30 pts; III-IV: 7 pts; BT: Age: 58.20 y; Gender: 9 male. CT: Age: 53.1 y; Gender: 5 male; 10 female.	Total RNA extracted with mirVana™ miRNA Isolation Kit (Applied Biosystems). RT with TaqMan MiRNA RT Kit (Applied Biosystems); qPCR with TaqMan MicroRNA Assays (Applied Biosystems). U6 as endogenous reference gene for normalitazion.	**diagnostic**. AUC = 0.955 (98.1% specificity and 100% sensitivity).
[77]	miR-30a-5p ME ↑	urine EVs	After collection, urine samplescentrifuged at 4000 rpm for 20 min at 4 °C and washed in PBS 1x. Lastly, pellets were frozen at − 80 °C.	retrospective/prospective; multicenter	**DC**: 53 RCC (ccRCC); 57 CT. **VC**: 171 RCC (ccRCC); 85 CT.	**DC**: RCC: Age: 61 y; TNM: I-II: 30 pts; III-IV: 23 pts; Fuhrman grade: G1-G2: 28 pts; G3-G4: 25 pts. CT: Age: 49 y. **VC**: RCC: Age: 66 y; TNM: I-II: 129 pts; III-IV: 42 pts; Fuhrman grade: G1-G2: 54 pts; G3-G4: 8 pts; CT: Age: 55 y.	DNA extracted with phenol-chloroform method; bisulfite modification with EZ DNA Methylation-Gold™ Kit (Zymo Research); pre-amplification step; quantitative methylation-specific PCR (SsoAdvanced™ PreAmp Supermix, Bio-Rad) with Quantitative Methylation-specific PCR assays with Xpert Fast SYBR (Grisp). β-Actin as endogenous reference gene for normalitazion.	**diagnostic and prognostic**. AUC = 0.684, with 83% sensitivity and 53% specificity (testing cohort); AUC = 0.67 with 63% sensitivity and 67% specificity (validation cohorts).
[78]	miR-210-3p ↑	urine EVs	Urine samples were frozen within 30 min from collection and stored at −80 °C until RNA extraction.	prospective; monocenter	DC: 21 RCC (ccRCC).	**DC**: RCC: Age: 63 y; Gender: 13 male; TNM: I-II: 16 pts; III-IV: 5 pts. Fuhrman grade: G1-G2: 10 pts; G3-G4: 11 pts.	Total RNA extracted with miRNAeasy serum/plasma kit (Qiagen). RT with miScript II RT kit (Qiagen). qPCR with miScript Primer Assay (Qiagen) with miScript SYBR Green PCR kit (Qiagen). Cel-miR-39 as exogenous reference gene for normalization.	**diagnostic and prognostic**.*p* <0.05.
[79]	miR-498 ↑, miR-183 ↑, miR-205 ↑, miR-31 ↑	urine EVs	Urine samples collected and stored frozen at −20 °C until use.	prospective; monocenter	**DC**: 31 RCC (10 ccRCC, 5 chRCC, 6 pRCC); 7 BT: ; CT: 5.	Fuhrman grade-ISUP: only grade I-II.	MiRNA extracted with miRNeasy kit (Qiagen). RT with random primers and SuperScript III RT (Invitrogen); qPCR with mirVana qPCR primer set for 5S ribosomal RNA used for normalization.	**diagnostic**. *p* <0.001.

DC = diagnostic cohort; VC = validation cohort; DP = diagnostic phase; VP: validation phase; AUC = area under curve; CI = confidence interval; PFS = progression-free survival; CSS = cancer-specific survival; DSS = disease-specific survival; OS = overall survival; HR = hazard ratio.

## Data Availability

Not applicable.
